# *Bacillus megaterium* strains derived from water and soil exhibit differential responses to the herbicide mesotrione

**DOI:** 10.1371/journal.pone.0196166

**Published:** 2018-04-25

**Authors:** Tatiane Dobrzanski, Fernanda Gravina, Bruna Steckling, Luiz R. Olchanheski, Ricardo F. Sprenger, Bruno C. Espírito Santo, Carolina W. Galvão, Péricles M. Reche, Rosilene A. Prestes, Sônia A. V. Pileggi, Francinete R. Campos, Ricardo A. Azevedo, Michael J. Sadowsky, Flávio L. Beltrame, Marcos Pileggi

**Affiliations:** 1 Laboratório de Microbiologia Ambiental, Setor de Ciências Biológicas e da Saúde, Departamento de Biologia Estrutural, Molecular e Genética, Universidade Estadual de Ponta Grossa, Ponta Grossa, Paraná, Brazil; 2 Laboratório de Biologia Molecular e Ecologia Microbiana, Instituto de Ciências Biomédicas, Departamento de Microbiologia, Universidade de São Paulo, São Paulo, São Paulo, Brazil; 3 Separare - Núcleo de Cromatografia, Departamento de Química, Universidade Federal de São Carlos, São Carlos, São Paulo, Brazil; 4 Laboratório de Biotecnologia Microbiana, Departamento de Biotecnologia, Genética e Biologia Celular, Universidade Estadual de Maringá, Maringá, Paraná, Brazil; 5 Laboratório de Biologia Molecular Microbiana, Setor de Ciências Biológicas e da Saúde, Departamento de Biologia Estrutural, Molecular e Genética, Universidade Estadual de Ponta Grossa, Ponta Grossa, Paraná, Brazil; 6 Laboratório de Pesquisa em Recursos Hídricos, Setor de Ciências Biológicas e da Saúde, Departamento de Enfermagem e Saúde Pública, Universidade Estadual de Ponta Grossa, Ponta Grossa, Paraná, Brazil; 7 Departamento Acadêmico, *Campus* Ponta Grossa, Universidade Tecnológica Federal do Paraná, UTFPR, Campus Ponta Grossa, Ponta Grossa, Paraná, Brazil; 8 Laboratório de Biociências e Espectrometria de Massas, Departamento de Farmácia, Universidade Federal do Paraná, Curitiba, Paraná, Brazil; 9 Departamento de Genética, Escola Superior de Agricultura Luiz de Queiroz, Universidade de São Paulo, Piracicaba, São Paulo, Brazil; 10 Department of Soil, Water, and Climate, and The Biotechnology Institute, University of Minnesota, Saint Paul, Minnesota, United States of America; 11 Laboratório de Fitoterapia, Tecnologia e Química de Produtos Naturais, Departamento de Ciências Farmacêuticas, Setor de Ciências Biológicas e da Saúde, Universidade Estadual de Ponta Grossa, Ponta Grossa, Paraná, Brazil; Universite Paris-Sud, FRANCE

## Abstract

The intense use of herbicides for weed control in agriculture causes selection pressure on soil microbiota and water ecosystems, possibly resulting in changes to microbial processes, such as biogeochemical cycles. These xenobiotics may increase the production of reactive oxygen species and consequently affect the survival of microorganisms, which need to develop strategies to adapt to these conditions and maintain their ecological functionality. This study analyzed the adaptive responses of bacterial isolates belonging to the same species, originating from two different environments (water and soil), and subjected to selection pressure by herbicides. The effects of herbicide Callisto and its active ingredient, mesotrione, induced different adaptation strategies on the cellular, enzymatic, and structural systems of two *Bacillus megaterium* isolates obtained from these environments. The lipid saturation patterns observed may have affected membrane permeability in response to this herbicide. Moreover, this may have led to different levels of responses involving superoxide dismutase and catalase activities, and enzyme polymorphisms. Due to these response systems, the strain isolated from water exhibited higher growth rates than did the soil strain, in evaluations made in oligotrophic culture media, which would be more like that found in semi-pristine aquatic environments. The influence of the intracellular oxidizing environments, which changed the mode of degradation of mesotrione in our experimental model and produced different metabolites, can also be observed in soil and water at sites related to agriculture. Since the different metabolites may present different levels of toxicity, we suggest that this fact should be considered in studies on the fate of agrochemicals in different environments.

## Introduction

The herbicide Callisto (Syngenta, Basel, Switzerland) is used for pre- and post-emergent control of broadleaf weeds in maize crops. Its active ingredient mesotrione {2- (4-methylsulfonyl-2-nitrobenzoyl)-1,3-cyclohexanedione} belongs to triketone group, and its mechanism of action is associated with the inhibition of the enzyme 4-hydroxyphenylpyruvate dioxygenase (HPPD, EC 1.13.11.27), which is involved in the biosynthesis of carotenoids in plants [1, 2].

Few studies have evaluated the biodegradation of mesotrione [3, 4] and the relationship of this herbicide with the generation of ROS and bacterial damage [5, 6]. Mesotrione transformed by *Bacillus* sp. 3B6 [3], produced the metabolites 4-methylsulfonyl-2-nitrobenzoic acid (MNBA) and 2-amino-4-methylsulfonyl benzoic acid (AMBA), which was considered highly toxic relative to the parent compound [2, 7, 8]. However, these authors proposed that this herbicide is used as a sole carbon source by *Bacillus* strain 3B6. In contrast, Pileggi et al. [4] suggested that mesotrione, at environmentally relevant concentrations, was transformed by *Pantoea ananatis* to avoid the toxic effects of this herbicide, rather than a source of carbon.

Tolerant and/or degrading microorganisms may have distinct metabolic responses when exposed to herbicides, including an increased generation of reactive oxygen species (ROS) [9, 10, 11]. ROS, such as superoxide (O_2_^-^), hydrogen peroxide (H_2_O_2_), and the hydroxyl radical ^●^OH, are generated continuously in cells under aerobic conditions [12, 13]. When in contact with xenobiotics such as herbicides, metabolic processes can trigger an intracellular redox imbalance with an increase in ROS to levels that exceed the cell defense capability, resulting in oxidative stress [14]. Oxidative damage can result in DNA mutation, protein inactivation and denaturation, lipid peroxidation, decreased cellular growth, or cell death [15, 16, 17, 18].

To minimize the harmful effects of ROS, aerobic organisms have developed antioxidant defense system that helps decreasing ROS production [16, 19, 20]. Among the enzymatic defense mechanisms, superoxide dismutase (SOD, EC 1.15.1.1) is responsible for the initial stage of cellular detoxification and is involved in the dismutation of O_2_^-^ to generate H_2_O_2_ and O_2_ [16, 19]. The H_2_O_2_ can subsequently diffuse in the cellular environment and cause damage; thus, its effects are lowered by the activities of catalase (CAT, EC 1.11.1.6) and other peroxidases such as alkyl hydroperoxide reductase (Ahp, EC 1.11.1.15). CAT converts H_2_O_2_ into O_2_ and H_2_O [21, 22], which are not toxic. These cellular defense responses are important for survival to the oxidative stress condition caused by herbicides and other xenobiotics [13, 23].

Herbicides applied in agriculture systems can result in a strong selective pressure not only to the soil microbiota, but also to microbes present in waterways located near the application area. This is mainly due to the fact that these chemicals undergo processes such as leaching and sorption and consequently contaminate surface and ground waters [24, 25]. Therefore, the selective pressure caused by herbicides on the microbiota in agricultural soils may also occur to those in waterways, despite fundamental differences in these environments.

Studies on the adaptations and responses of microbiota from different environments exposed to herbicides can improve the understanding, and the practical use, of bioremediation processes to mitigate problems arising from the intensive use of pesticides. In the present study, we analyzed the response of two phylogenetically close bacterial isolates, *Bacillus megaterium* species strains CCT 7729 isolated from water (NCBI accession number KR057954), and CCT 7730 (NCBI accession number KR057955), isolated from soil, to the herbicide mesotrione. Our objective was to understand the variation in response to Callisto, and its active ingredient mesotrione, presented by two bacterial strains of the same species, isolated from soil and water environments.

## Material and methods

### Chemicals

The commercial product Callisto (Syngenta Crop Protection, Greensboro, NC, USA, containing 48% of active ingredient mesotrione), and mesotrione (92% purity diluted in acetonitrile in a final concentration of 0.67 mg/mL; Syngenta Crop Protection) were used for microbiological assays. The concentration of mesotrione used was 0.04 mM, which was equivalent to the application dose of the herbicide in the field, according to the instructions published by the manufacturer. An analytical standard of mesotrione (99% purity, Pestanal, Sigma Aldrich) was used in high-performance liquid chromatography (HPLC-DAD) and liquid chromatography coupled with mass spectrometry (LC-MS/MS) analysis.

### Sample collection and bacterial isolation

Soil samples from a maize field and water samples from a pond close to this farm were collected in the Capão do Cipó farm (Experimental Station of the ABC Research Foundation for Assistance and Technical Information in Agriculture), under permit issued by Senio Jose Napoli Prestes, phytopathologist leader. The site is located at 24°51’28”.7 S and 49°55’57”.7 W in Castro, Paraná state, Brazil. The herbicide Callisto was applied one month before crop harvesting and soil sampling. Serial dilutions of soil and water were used to obtain bacterial isolates. Bacterial isolates from each environment (soil and water) were selected for study, due to their capacity to grow in culture media containing Callisto or mesotrione. The following culture medium was used: mineral medium (MM) (10 mM potassium phosphate buffer, pH 7.0, containing 3 gL^-1^ of NaNO_3_, 0.5 gL^-1^ of MgSO_4_, 0.5 gL^-1^ of KCl, 0.01 gL^-1^ of FeSO4, 0.04 gL^-1^ of CaCl_2_, 0.001 gL^-1^ of MnSO_4_ and 20 gL^-1^ of glucose); MM combined with 0.04 mM mesotrione active ingredient (MMM); and MM combined with Callisto equivalent to 0.04 mM mesotrione (MMC). All experiments were performed in triplicate.

### Isolation of strains and molecular identification

Bacteria were obtained by inoculating soil or water samples into Luria Broth (LB) medium (10 gL^-1^ tryptone, 5 gL^-1^ yeast extract, and 10 gL^-1^ NaCl) and incubation at 30 °C, with stirring at 120 rpm, until they reached the exponential growth phase. Cultures were streaked onto LB agar medium, and single, pure, colonies were isolated by repeated streaking on the same medium. About 300 pure cultures were streaked onto LB agar plates containing Callisto at a mesotrione concentration at 0.04 mM. Colonies tolerant to the herbicide were frozen at -80 °C until used.

The identity of the most tolerant colonies from water and soil was determined by sequencing of the 16S rRNA gene. Total DNA was extracted using a Promega (Madison, WI) DNA isolation kit and 16S rRNA was amplified by PCR using the universal primers FD1 and RD1 [26]. DNA was sequenced on an ABI 3500 xL genetic analyzer (Applied Biosystems). The sequences were deposited in the GenBank database (http://www.ncbi.nlm.nih.gov) under accession numbers KR057954 and KR057955.

### Analysis of oxidative stress

For the evaluation of oxidative stress, enzymes, lipids, modifications of membrane lipids, products of degradation and cell viability, the bacterial isolates were cultured using the following protocol. Cultures (1800 mL) were grown in LB medium and incubated for 24 h at 30 °C in 1800 mL of LB liquid medium with stirring at 120 rpm. The cultured cells were centrifuged at 8,000×g at 4 °C and washed three times with phosphate buffer solution (PBS) (8 gL^-1^ NaCl, 0.20 gL^-1^ KCl, 1.44 gL^-1^ Na_2_HPO_4_, and 0.24 gL^-1^ KH_2_PO_4_). The final pellet was resuspended in 9 mL of MM and equally distributed in each medium (MM, MMM, and MMC), in triplicate. Culture were incubated at 30 °C at 120 rpm, and bacterial growth was evaluated in two periods: 3 h (corresponding to the mid-lag phase) and 14 h (mid-log phase), time periods that were used based on preliminary growth tests.

Aliquots of the MMM culture were collected at 0 h (control with 100% mesotrione), 3 h (mid-lag growth phase), and 14 h (mid-log growth phase) and centrifuged at 8.000×g and 4 °C; the supernatants were filtered through a PTFE membrane (0.22 μm) for analysis, in triplicate, by HPLC-DAD or LC-MS/MS as described by Pileggi et al. [4].

### Bacterial growth curves

The isolates were grown in 50 mL LB medium for 14 hours, until they reached the mid-log phase of growth. The cells were centrifuged at 8,000×g. The pellets were washed 3 times with PBS A buffer prior to inoculation into each medium (MM, MMM, and MMC) at an initial optical density (OD) of 0.05 (Spectrophotometer UV-1600, Pró-Análise, Curitiba, Brazil). Growth was evaluated at an absorbance of 600 nm. The cultures were diluted when reached an OD greater than 0.6 and the measures were obtained by multiplying by the dilution factor. Preliminary tests showed that the acetonitrile used for mesotrione dilution did not affect bacterial growth rates (data not shown).

### Cell viability

Bacterial cells were grown in the MM, MMM and MMC media, containing Callisto or mesotrione, for 3 and 14 h of incubation. Cells were recovered by centrifugation and diluted in PBS A buffer to remove herbicides residues. The 3 h cultures were diluted to 10^−5^, whereas the 14 h cultures were diluted to 10^−10^. Aliquots were plated, in triplicate, onto LB medium, without herbicide, containing 15 g L^-1^ agar and incubated at 30 °C for 24 h. The colony forming units (CFU) obtained after herbicides treatment were counted.

### Characterization of mesotrione degradation

Mesotrione degradation, observed at 3 and 14 h periods, was evaluated using a HPLC-DAD system (Waters, USA), containing a photo diode array detector at a wavelength of 254 nm, and a Nucleodur Gravity C_18_ column (150 x 4.6 mm, 5 μm) maintained at 20 °C. A 50μL sample was injected at a flow rate of 1.0 mL/min and the elution was performed in triplicate as described by Olchanheski et al. [5]. Empower 3 Chromatography data software was used to analyze the chromatograms. The analytical method was validated according the ICH guidelines [27]. Degradation was compared with 0 h control of each strain as well as non-inoculated controls.

### Identification of mesotrione metabolites

Metabolite produced by cultures grown in mesotrione-containing media were evaluated as described previously [4]. Briefly, an Esquire 6000 IT mass spectrometer, equipped with an electrospray ion (ESI) source (Bruker Daltonics, GmbH, Bremen, Germany) and equipped with a Shimadzu Proeminence LC system (Shimadzu, Kyoto, Japan) was used. The extracts samples were initially treated with formic acid and passed through a previously conditioned with formic acid HLB solid-phase extraction cartridge (SPE) (Waters). Chromatographic separation of compounds was obtained with a phenyl-hexyl Luna analytical column (150 mm × 4.6 mm, 10 μm) operated at 35 °C. The mobile phase consisted of 0.1% formic acid aqueous solution (solvent A) and acetonitrile (solvent B). The flow rate was maintained at 1.0 mL/min, and the gradient profile was as follows: 0 min– 5% (B); 5% to 26.5% in 18 min, 26.5% to 65% in 7 min, after that the percentage of B phase was changed to 95% and an isocratic run was maintained for 4 min before return to 5% (B) to equilibrate the column for a new injection. The sample injection volume was 10 μL. The analyses done using the ESI-IT-MS was run in the positive mode and spectra were acquired within a mass range of 50–400 Da. The ESI optimum conditions were a drying flow rate of 6 Lmin^-1^ at 300 °C using a nebulizing gas pressure of 30 psi. Experiments of MS/MS of ion of *m/z* 340 were evaluated in positive mode according the parameters: helium as collision gas (> 99% purity) and fragmentation amplitude was 1V, and smart parameters settings (SPS) were online mode, target mass (SPS) of ion of *m/z* 225. Data acquisition was carried out with the Data Analysis software (Bruker Daltonics, GmbH, Bremen, Germany).

### Hydrogen peroxide production

Hydrogen peroxide was quantified in a spectrophotometer at 390 nm by the iodine release method as previously described by Gravina et al. [11]. Protein extracts were obtained by the maceration of bacterial pellets in liquid nitrogen followed by homogenization in 1 mL of 0.1% TCA, and centrifuged at 8,000 × g for 15 min at 4 °C. The 200 μL reaction volumes contained 100 mg of protein extract, 200 μL of potassium phosphate buffer (pH 7.5), and 800 μL of 1 M potassium iodide. Reactions were incubated for 1 h on ice in the dark. The results were expressed in μmol/g of fresh weight [11].

### Determination of lipid peroxidation

Lipid peroxidation was determined spectrophotometrically by measuring the production of malondialdehyde acid (MDA), a metabolite reactive to 2-thiobarbituric acid, as described by Heath and Packer [28]. The MDA concentration was monitored at 535 nm (to read all lipids) and 600 nm (to read all lipids except MDA). The concentrations were calculated using an extinction coefficient of 155 mM.cm^-1^.

### Protein extraction

Proteins were extracted from the selected bacterial isolates during mid-lag and mid-log phases of growth. Bacterial cultures were incubated for 3 h and 14 h, and centrifuged at 8,000 × g at 4 °C. The pellet was macerated with liquid nitrogen and homogenized at a ratio of 1:10 (w/v) with 100 mM PBS B (pH 7.5) containing 14.520 g.L^-1^ K_2_HPO_4_, 2.260 g.L^-1^ KH_2_PO_4_, 0.372 g.L^-1^ EDTA, 0.462 g.L^-1^ DL-dithiothreitol, and 5% (w/w) polyvinylpyrrolidone (PVPP) [29]. The extract was centrifuged at 8,000 × g for 30 min and then the supernatant was collected. The protein concentration was determined using the Bradford method [30] with bovine serum albumin as the standard.

### Characterization of SOD isoforms

The SOD isoforms were assessed by using 12% non-denaturing PAGE as described by Azevedo et al. [31]. The gel was divided vertically into three parts and maintained in the dark. The first part was immersed in 100 mM potassium phosphate buffer (pH 7.8), the second part was immersed in 100 mL of 100 mM potassium phosphate buffer containing 2 mM KCN and 1 mM EDTA, and the third part was immersed in 100 mL of 100 mM potassium phosphate buffer with 5 mM H_2_O_2_ and 1 mM EDTA. The isoforms were classified as Mn-SOD if they were resistant to both inhibitors (KCN and H_2_O_2_), as Fe-SOD if they were resistant to KCN and inhibited by H_2_O_2_, and as Cu/Zn-SOD if they were inhibited by both inhibitors [31].

### SOD activity in non-denaturing PAGE

The SOD activities and isoforms were evaluated using 12% non-denaturing polyacrylamide gel electrophoresis (PAGE) as described by Cia et al. [32]. Electrophoresis was performed at a constant current of 15 mA for 3 h using 20 μg of each protein extract per well (Protein extraction section). At the end of electrophoresis, the gels were washed with deionized water and incubated in the dark at room temperature in 50 mM potassium phosphate buffer (pH 7.8) containing 1 mM ethylenediamine tetraacetic acid (EDTA), 0.05 mM riboflavin, 0.1 mM nitroblue tetrazolium (NBT), and 0.3% N,N,N’,N’-tetramethylethylenediamine (TEMED). The reaction mixture was discarded after 30 min. The gels were washed with deionized water and placed under a bright light for the identification of the bands [32].

### CAT activity in non-denaturing PAGE

CAT activity was determined using 12% non-denaturing PAGE as reported by Dourado et al. [33]. A current of 15 mA gel^−1^ was applied for 24 h at 4 °C and 15 μg of protein at sample were applied to the gel. Gels were washed with deionized water (3 times for 15 min) and incubated in 0.003% H_2_O_2_ for 10 min and developed in a 1% (w/v) FeCl_3_ and 1% (w/v) K_3_Fe(CN_6_) solution for 10 min.

### Modifications in membrane lipids

Modifications to membrane lipids was determined using the method of Prione et al. [6]. Bacterial cultures were centrifuged at 8,000 × g for 10 min. The pellet was macerated with liquid nitrogen. The samples were frozen at—80 °C and lyophilized for 15 h. A 4-mL volume of hexane was added to the lyophilized samples, and stirred for 48 hours at 60 rpm. At the end of 24 h, the supernatant was collected and the lipid fraction was extracted by hexane evaporation. A volume of 4 mL of hexane was again added to the samples, and stirred for 24 hours to obtain the total lipid extract. The saturation of membrane lipids was analyzed by Fourier Transform Infrared Spectroscopy (FTIR), IRPrestige-21 with diffuse reflectance accessory, DRS-8000/Shimadzu (Kyoto, Japan), at a wavenumber of 400–4000 cm^-1^, with a resolution of 2 cm^-1^, using 100 mg of potassium bromide (KBr) and 0.001 g of lipid extract for the assembly of the chip for analysis.

### Statistical analysis

The data obtained from growth curves, cell viability assays, and quantification of MDA and H_2_O_2_ were analyzed by using two-way ANOVA in the Stata 12 software package, followed by Bartlett’s test for inequality of population variances, and Bonferroni’s correction to compare the variables between the treatments, growth periods, and strains. Statistical analyses were performed with three completely random repetitions of each treatment, and the significance criterion was p < 0.05.

For the analysis of lipid saturation, Multivariate Principal Component Analysis (PCA) and regression by Partial Least Squares (PLS) were applied using the Pirouette software version 4.0 (Bothell, WA, USA). Lipid profiles were constructed from FTIR data and evaluated by PCA and PLS. A matrix of 36 x 4000 (samples x wavenumber range 4000–400 cm^-1^) was constructed. For the PLS the matrix was correlated with the dependent variables (time, MDA, peroxide and cell viability). Several pre-processes (mean-center, autoscale and variance scale) and treatments (first and second derivatives and multiplicative scatter correction—MSC) were used for the multivariate analyses.

## Results

### Growth of strains in media containing Callisto and mesotrione

**Fig 1:**
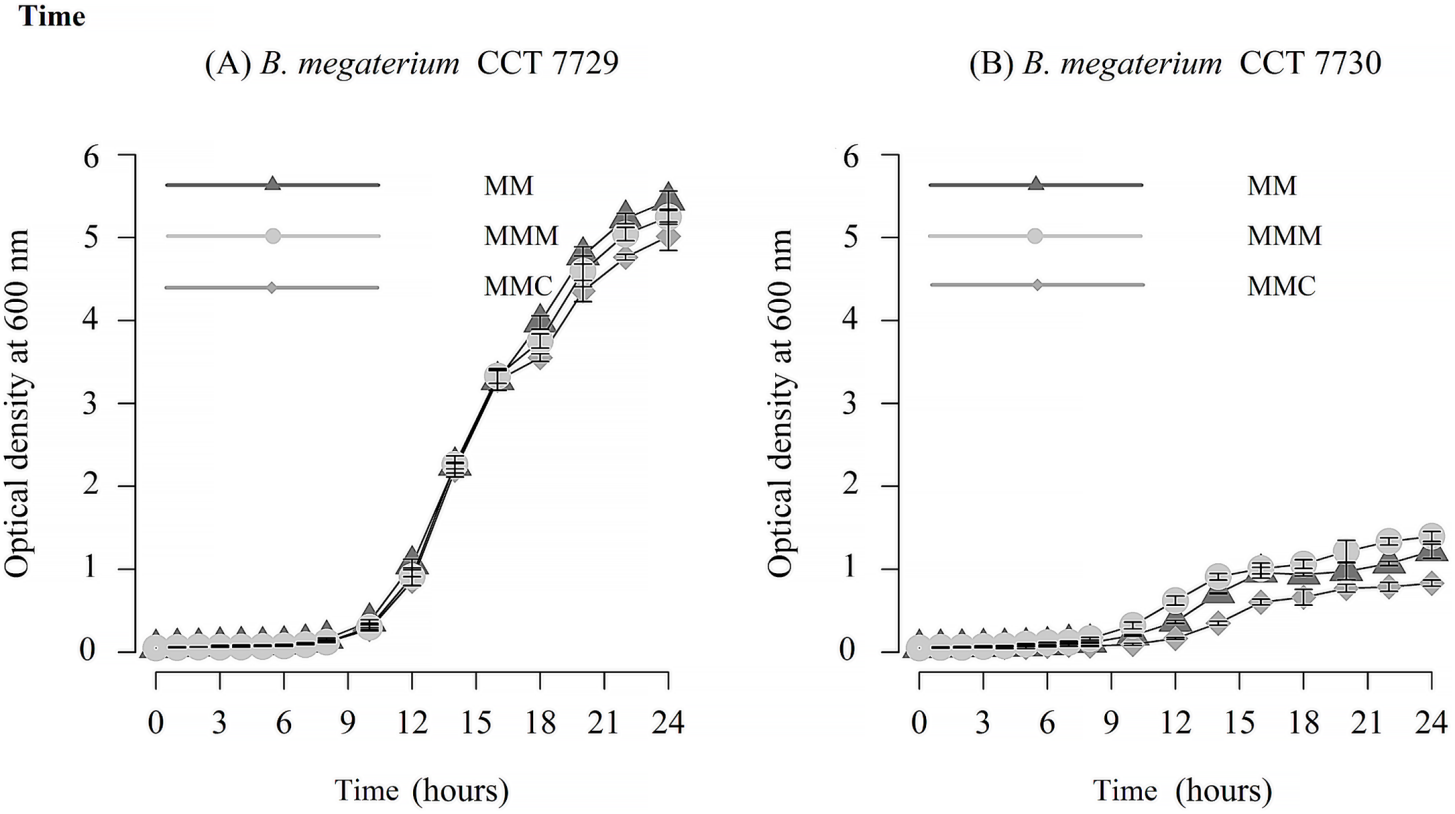
Growth curve of the isolates (A) *B*. *megaterium* CCT 7729 and (B) *B*. *megaterium* CCT 7730. Legend: MM, MMM, and MMC indicate mineral medium, mineral medium with mesotrione, and mineral medium with Callisto, respectively. The bars represent the standard errors on means.

Analysis of near full-length DNA sequence of 16S rRNA indicated that strains CCT 7729 and CCT 7730 had 99% and 100% sequence identity, respectively, with *B*. *megaterium* strain JX285 (accession CP018874.1). Results in Fig 1 show that the strains appeared to be in lag phase after 3 h of incubation and in mid-log of growth by 14 h. Therefore, these two periods were selected for the analysis of the behavior of isolates CCT 7729 and CCT 7730 exposed to mesotrione active ingredient and Callisto (Fig 1).

Both isolates exhibited lower growth rates in the MMC medium compared with MM medium (S1 File, section 1.1 and 1.2). In contrast to strain CCT 7729, strain CCT 7730 exhibited higher growth rates in the MMM medium (S1 File, section 1.2). Nevertheless, strain CCT 7729 strain showed growth rates higher than the CCT 7730 strain, in the three-culturing media tested (S1 File, section 1.3), resulting in an approximately a 5-fold increase in biomass from water to soil strain (Fig 1).

### Toxicity and rates of cell damage caused by Callisto and mesotrione

The Bartlett’s Test for inequality of population variances presented p-value less than 0.05 for several viability experiments, suggesting that the variances are not homogeneous (S2 File). In this case, we did not use the ANOVA tests, which was inappropriate in these instances.

**Fig 2:**
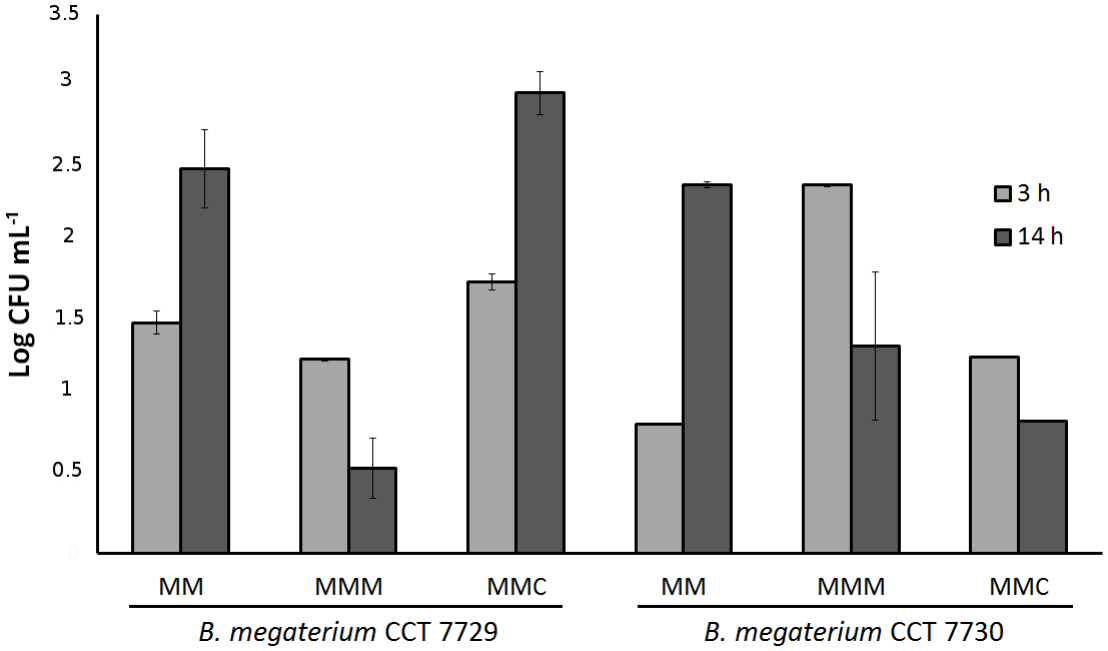
Cell viability of *B*. *megaterium* isolates CCT 7729 and CCT 7730 after 3 h and 14 h of growth in MM, MMM and MMC media. Data were analyzed using two-way ANOVA followed by Bonferroni´s correction, *p < 0.05. The bars represent the standard errors on means.

The number of viable cells in the control and herbicide treatment groups is shown in Fig 2. Generally, the strains behaved differently during treatment with mesotrione or Callisto [4, 5, 6]. For strain CCT 7729, treatment with mesotrione resulted in a large decrease in viable cell numbers by 14 h. In contrast, treatment with Callisto resulted in increased growth by 14 h of incubation. Relative to growth seen with the 3 h culture growth of strain CCT7729, growth in mesotrione resulted in a decrease in viable cells, while there was an increase in cell number following growth in Callisto. Both strains grew equally well after 14 h of growth in control cultures. Results of this study show that there are differential responses to formulated vs non-formulated herbicide and that strain CCT 7730 was more sensitive to Callisto than strain CCT 7729.

**Fig 3:**
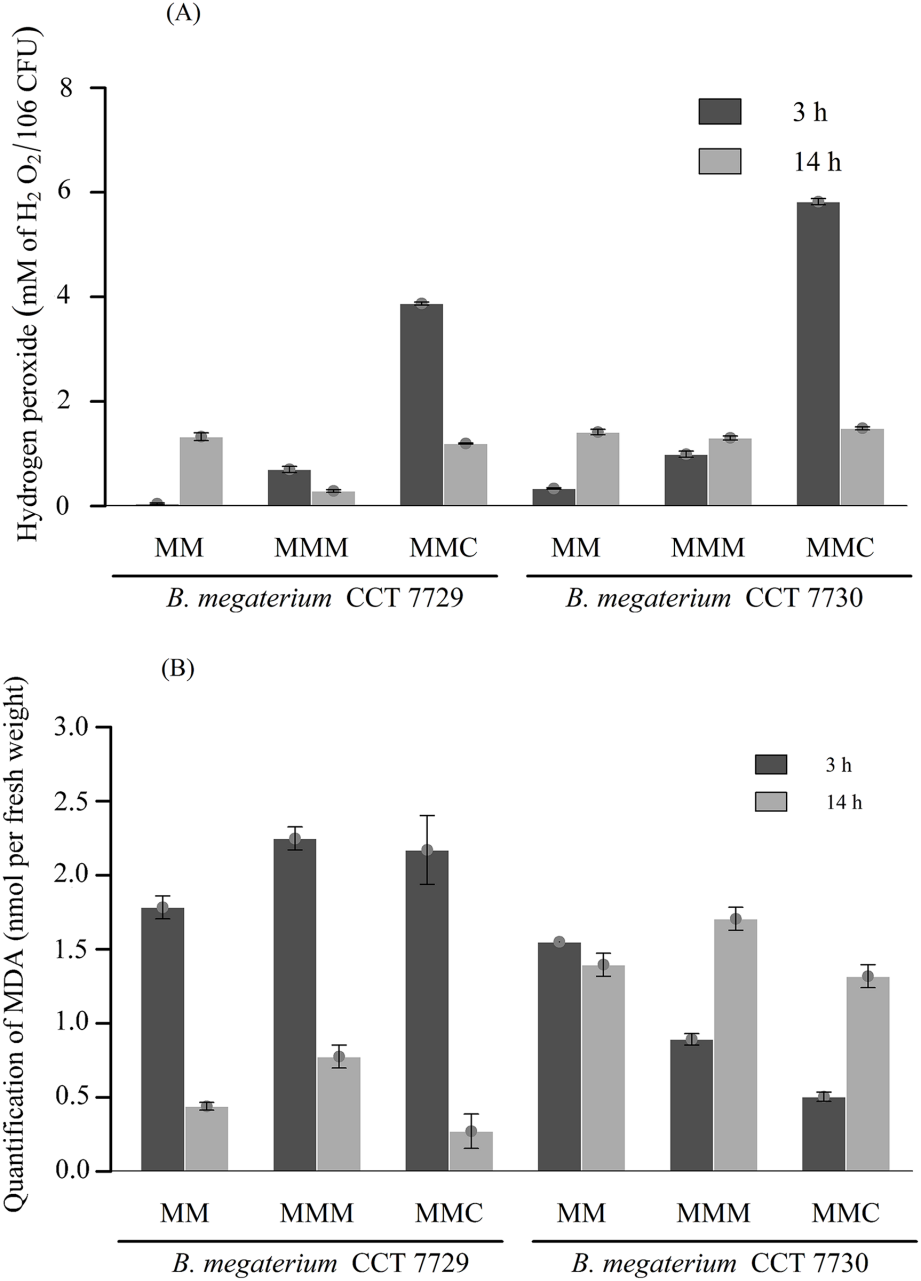
(A) Quantification of H_2_O_2_ levels and (B) MDA in the *B*. *megaterium* isolates CCT 7729 and CCT 7730 after 3 h and 14 h periods in the MM, MMM, and MMC media. Data were analyzed using two-way ANOVA followed by Bonferroni’s correction, *p < 0.05. The bars represent the standard errors on means.

The quantification of H_2_O_2_ and MDA in the two *B*. *megaterium* isolates is shown in Fig 3a and 3b, respectively. The peroxide levels after treatment with Callisto, at lag phase, were higher for both strains (Fig 3a), but with significant reduction at mid log phase (S3 File, Section 3.1 and Section 3.2). By 14 h of growth, both treatment groups and the control had similar amounts of H_2_O_2_ and a similar pattern was seen for the control cultures.

The two isolates exhibited contrasting behaviors regarding MDA levels after treatment with the herbicides (Fig 3b). While for *B*. *megaterium* CCT 7729 the MDA rates at lag phase were higher than at mid log phase (S4 File, Section 4.1), the opposite situation was seen for strain CCT 7730 (S4 File, Section 4.2). A negative correlation between the MDA levels (Fig 3b) and cell viability (Fig 2) was observed only for *B*. *megaterium* strain CCT 7729.

### Degradation of mesotrione

The HPLC-DAD analysis revealed at 0 h of incubation for both bacteria, only one peak at R_t_ = 22.9 min correspondent to the presence of all amount of the herbicide added in the media. However, after 3 h and 14 h of incubation of bacteria strains was quantified 91.6% and 50.3% of mesotrione for *B*. *megaterium* CCT 7729, and 84.8% and 37.5% of mesotrione for *B*. *megaterium* CCT 7730, respectively, were quantified (data not shown).

In LC-MS/MS data analysis, positive ESI mode, evidenced that both bacteria strains (*B*. *megaterium* CCT 7729 and *B*. *megaterium* CCT 7730) can degrade the added mesotrione after 24 h of incubation (S1 Fig, panels A and B, respectively). When herbicide was added to the media at 0 h, a mean peak at R_t_ = 22.9 min was observed that correspond to mesotrione molecule protonated at *m/z* 340 [M+H]^+^ (S2 Fig). The MS/MS experiment of this ion yielded the ion fragment of *m/z* 228 correspondent to acylium ion [M+H-C_6_H_6_O_2_]^+^ (S2 Fig, panel D) [7].

However, after 24 h, the main peak of mesotrione (R_t_ = 22.9 min) decreased drastically for both bacteria strains evaluated, and different chromatographic profiles were observed (S1 Fig). The *B*. *megaterium* CCT 7729 data analysis showed a less complex metabolite profile, presenting only two new peaks with R_t_ = 11.6 min and 17.8 min (S3 Fig, panel A). In the MS spectrum of the peak at R_t_ = 17.8 min it was possible to observe an ion with a *m/z* 294. This was likely due to the loss of the nitro group of mesotrione to form this derivative compound (S3 Fig, panel C). In addition, it was observed in R_t_ = 11.6 min the ion at *m/z* 213 that was not identified in this study (data not shown).

On the other hand, for *B*. *megaterium* CCT 7730, the chromatographic fingerprinting of the metabolites presented a more complex profile, with several peaks related with different metabolites that were not identified in this study (S1 Fig, panel B).

### Antioxidant enzymes

#### SOD

**Fig 4:**
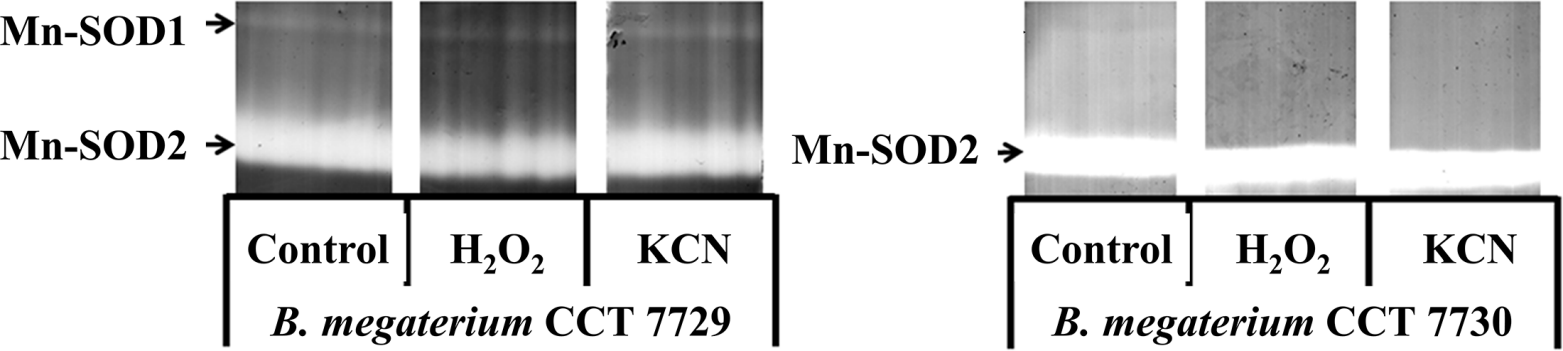
Characterization of the SOD isoforms in *B*. *megaterium* isolates CCT 7729 and CCT 7730.

Two Mn-SOD isoforms were found in *B*. *megaterium* CCT 7729, but only one was found in *B*. *megaterium* strain CCT 7730 (Fig 4).

**Fig 5:**
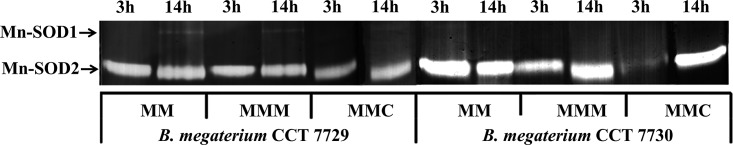
SOD-PAGE of samples from *B*. *megaterium* isolates CCT 7729 and CCT 7730 after 3 and 14 h of incubation in the control (MM) and treatment (MMM and MMC) media.

The SOD activity in the *B*. *megaterium* isolates CCT 7729 and CCT 7730 determined by PAGE is shown in Fig 5. The specific activity of Mn-SOD1 isoform was observed only in CCT 7729, at the log phase of growing in the control and mesotrione treatment. The Mn-SOD2 isoform activity was present and with high activity in all times and treatments of CCT 7730, except for 3 h MMC treatment.

#### CAT

**Fig 6:**
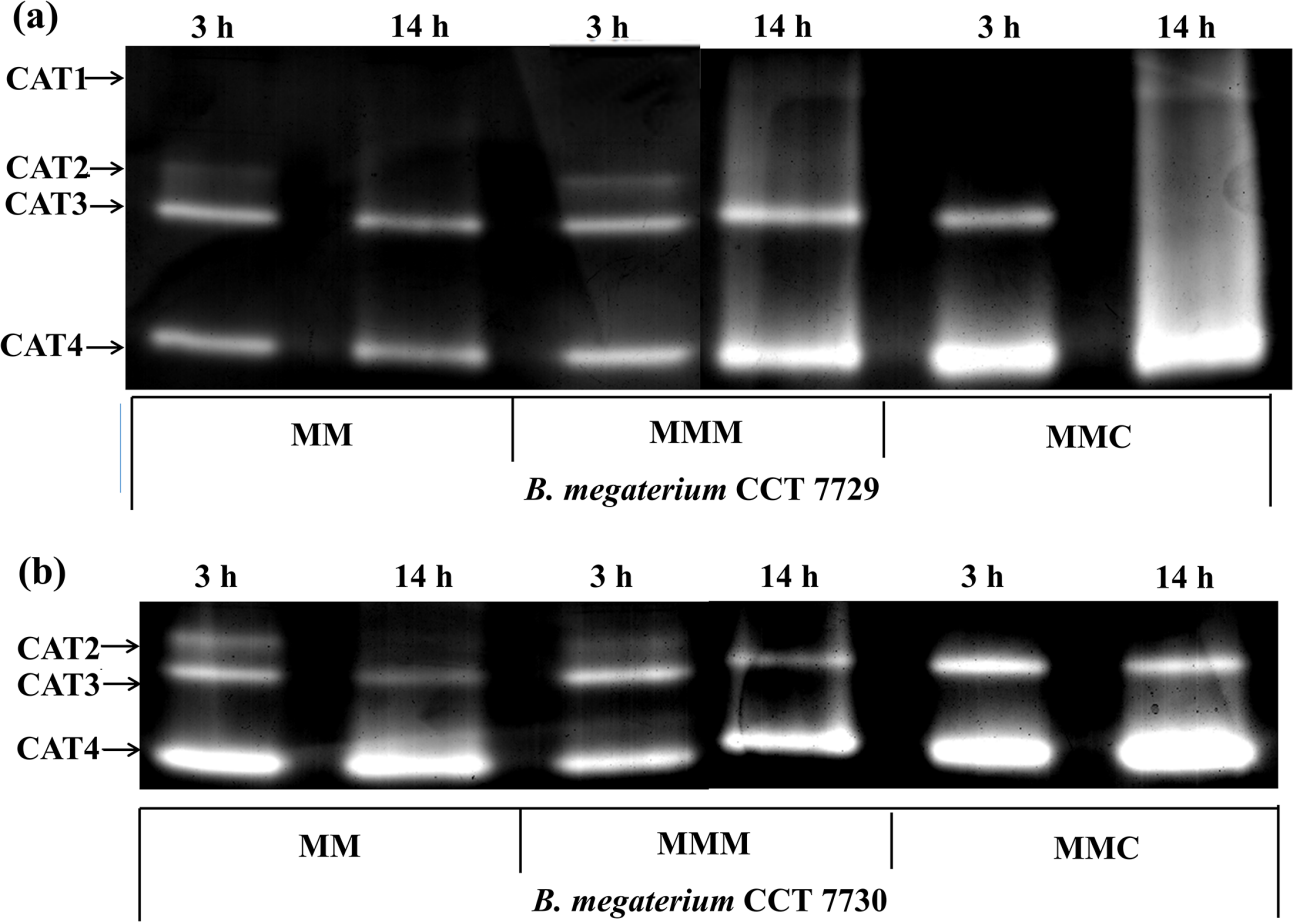
CAT-PAGE of *B*. *megaterium* CCT 7729 (a) and *B*. *megaterium* CCT 7730 (b) after 3 and 14 h of growth in the control medium (MM) and treatment media (MMM and MMC).

The activity of the antioxidant enzyme CAT was evaluated by PAGE (Fig 6). Four isoforms were found for CCT 7729 strain, and three for CCT 7730 (Fig 6). The isoforms with the highest global activity in both strains, called CAT3 and CAT4, possibly correspond to the HPII and HPI isoenzymes. Two additional isoenzymes were found in this work: CAT2, for both strains, and CAT1, exclusive for CCT 7729.

CAT1 was found exclusively in the CCT 7729 strain, with the highest activity at log phase in the mesotrione and Callisto treatments. The CAT2 was found in both strains, with the higher activity more restricted to the lag phase and in the CCT 7730 strain. The most interesting fact was that this isoform was not expressed in the Callisto treatments. The CAT3, which should correspond to well know HPII enzyme, presents greater activities in CCT 7730 at lag phase. The most highlight datum is the absence of activity in log phase in the Callisto treatment in the CCT 7729 strain. Nevertheless, the CAT4 isoform, which may correspond to HPI, also shows a higher activity in CCT 7730 than CCT 7729, especially in the Callisto treatments, but at log phase.

### Lipid saturation

**Fig 7:**
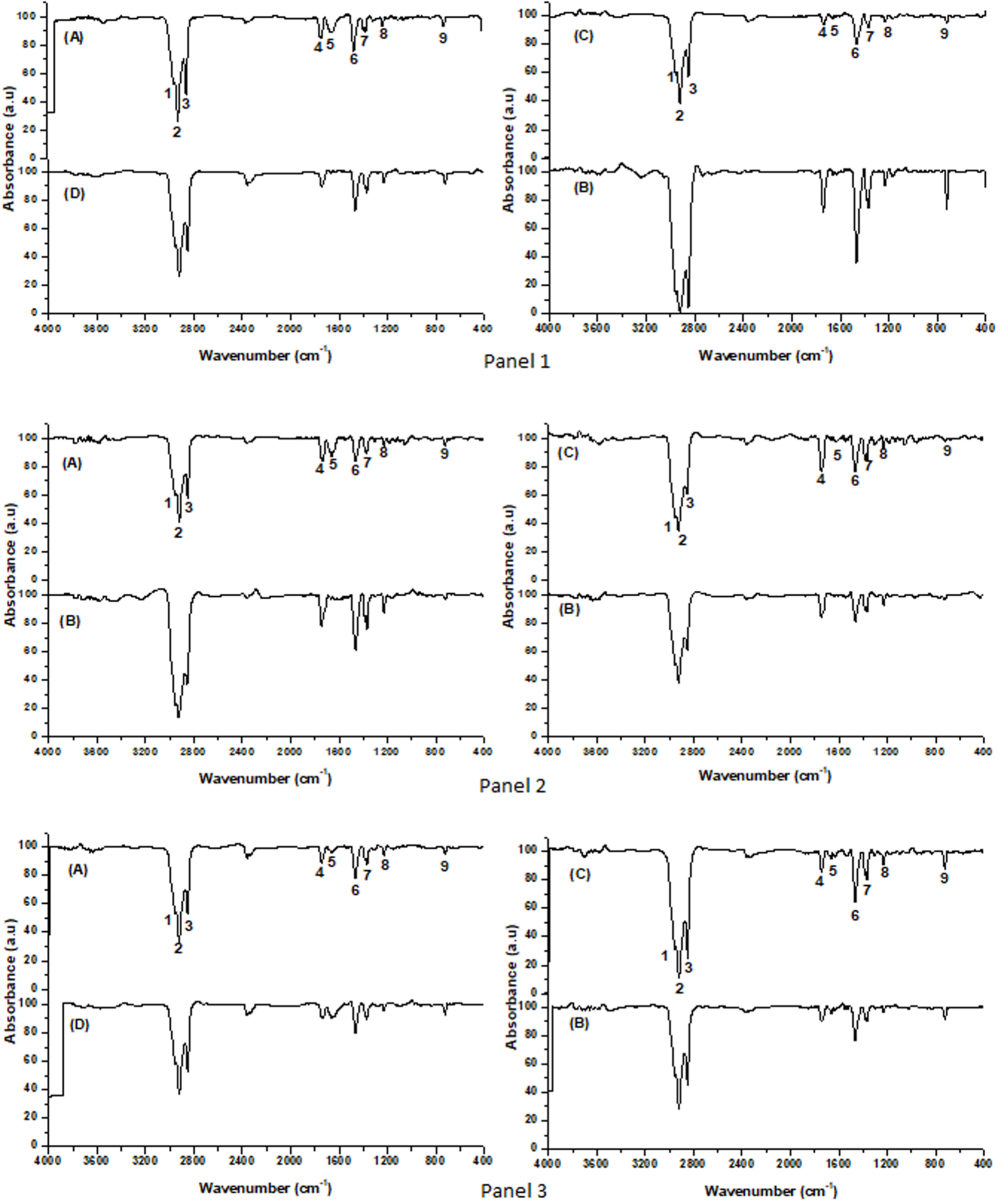
Infrared spectrum profile of the lipid extract obtained after incubation on MM (Panel 1); MMM (Panel 2) and MMC (Panel 3) of *B*. *megaterium* CCT 7729, (A) 3 h, (B) 14 h and *B*. *megaterium* CCT 7730, (C) 3 h (D) 14 h (FTIR analysis with scans from 4,000 cm^-1^ to 400 cm^-1^). Bands 1 e 2 correspond to (CH_2_); 3 to (CH_3)_; 4 to (C = O); 5 to (-C = C); 6 to (CH_2_); 7 to (CH_3_); 8 to (C-O) and to 9 (CH).

The lipid saturation of membrane in *B*. *megaterium* CCT 7729 and CCT 7730 under the effects of mesotrione and Callisto was analyzed by FTIR. The spectra showed qualitative and quantitative characteristic bands, which differs between the bacterial strains treated with mesotrione (Fig 7) and with treatment with Callisto and control (S1 Table). A typical band corresponding to alkanes indicating C-H_3_ stretching (2956 and 2872 cm^-1^) and C-H_2_ bonds (2920 and 2850 cm^-1^); and a band corresponding to C = O stretching (1,750 cm^-1^) characteristic of esters [34]. In addition to the unsaturation of fatty acids in the carbonyl region, the absorption band C = C (1650 cm^-1^) can also be observed in some samples. The absorption between 1477–1452 cm^-1^ and 725 cm-1 may be due to the asymmetric stretching and deformation of the C-H of methylene groups of the fatty acids, which are characteristic of long chain hydrocarbons containing (-CH_2_-)n. The 1380 to 1350 cm^-1^ region is the -CH_3_ group indicative of the isopropyl group [35]. At approximately 1229 cm^-1^ lies the C-O group stretching vibration region of esters [36].

The FTIR spectra of the evaluated samples showed the same saturation profile, but there was difference in some samples in the C = C absorption band at 1650 cm^-1^, and in the bands intensities. The averages of the absorption areas from the functional groups were identified by the FTIR spectra (S1 Table).

The results of PCA analysis of the FTIR spectra are show at (S4 Fig). The figure shows that the strains could be divided into two groups based of spectral analyses. With pre-processing, the first derivative treatment explained 99.11% of information with ten Main Components (CP). This exploratory analysis of the FTIR data showed that there are differences between the two strains and the lipid profile and incubation period.

Regression analysis done using the PLS model of FTIR data from strains *B*. *megaterium* CCT 7729 and *B*. *megaterium* CCT 7730, with time, the concentrations of MDA, peroxide and viability, revealed that several validation correlation coefficients (r_val_) and calibration (r_cal_) were obtained for these variables. However, the most significant correlation coefficients occurred when each strain was analyzed individually. The significant correlation coefficients for the time variable were r_val_ = 0.85 and r_cal_ = 0.99 (*B*. *megaterium* CCT 7729) and r_val_ = -0.84 and r_cal_ = 0.71 (*B*. *megaterium* CCT 7730) obtained through mean center preprocessing and Multiplicative Treatment Scatter Correction (MSC) and MSC preprocessing and second derivative, respectively.

The variables that presented the best calibration models for the two strains were the lipid saturation pattern and the incubation times. The variables that obtained significant values for the models in *B*. *megaterium* CCT 7729 strain were the regions of the lipid spectrum ranged from 705–750; 1209–1527 (C-O); 1697–1780 (C = C); 2789–3280 (C-H_3_ and C-H_2_) cm^-1^, indicating the prevalence of unsaturated lipids and, therefore, higher levels of cellular membranes fluidity. Nevertheless, the significant values for *B*. *megaterium* CCT 7730 strain were at regions of 950 to 1500 (C-H) cm^-1^, indicating the prevalence of saturated lipids and lower levels of cellular membranes fluidity.

The regression using the PLS model of the FTIR data of CCT 7729 and CCT 7730 with several validation correlation coefficients (rval) and calibration (rcal) is shown in S5 Fig. The vector relates the incubation time (dependent variable) to the concentration of the functional groups related to the lipid profile. The Y axis indicates the weight of variables distant from the zero axis above (positive) and below (negative) that influenced the lipid profile. Thus, the variables that presented the best calibration models for the lipid saturation profile of the two strains are different over time, according to the regression graph. The variables with significant values for the models were the regions of the spectrum of 950 to 1500 cm^-1^ for CCT 7729 strain and 705–750; 1209–1527; 1697–1780; 2789–328 cm^-1^ for CCT 7730 strain.

## Discussion

### Tolerance of strains to Callisto and mesotrione

Microorganisms exposed to environmental stresses undergo rapid changes in cellular processes to adapt and ensure survival. One of the mechanisms used to induce tolerance is the ability to modulate growth to a greater or lesser extent [37, 38]. The analysis of the bacterial growth curves of CCT 7729 and CCT 7730 strains, exposed to mesotrione active ingredient and Callisto (Fig 1), suggested that the afore mentioned strategies might be used by these isolates.

### Toxicity and rates of cell damage caused by Callisto and mesotrione

Callisto is comprised of 48% mesotrione and 52% of other ingredients, including poly (oxy-1-2-ethanediol), 1-octanol, and alpha-isodecyl-omega-hydroxy-phosphate, which increase the stability and enhance the effects of the herbicide [39]. These adjuvants may affect the solubility and sorption of the herbicide in the soil and consequently its bioavailability for microbial degradation [40]. Callisto may have direct effects on bacteria and fungi, including the inhibition of the colonization of some phototrophic microbial communities [39], and may negatively impact the chlorophyll concentration, genetic structure, and diversity of cyanobacteria in the soil [40]. Callisto toxicity was also observed in *P*. *ananatis*, which failed to grow in high concentrations of this herbicide (more than 100-fold the field dose) [4]. Callisto is, indeed, toxic for the *B*. *megaterium* strains studied in this work, once the growth rates in the MMC medium are statistically lower compared with MM and MMM in both strains (Fig 1).

Nevertheless, strain CCT 7729 showed an approximately 5-fold increase in biomass relative to strain CCT 7730, in the three-culturing media tested (Fig 1). Besides that, the former strain, isolated from water, had the best viability performance at Callisto treatment (Fig 2). Similar results for high Callisto viability rates at log phases were obtained for *P*. *ananatis*, also isolated from water [6]. It is possible that these rates represent the better adaptability of CCT 7729 strain to oligotrophic conditions since MM has less nutrients than LB, just as water has less nutrients than soil. The CCT 7730 strain, isolated from nutrient rich soil, would likely adapt less to the nutrient defined condition found in MM, than strain CCT 7729. High levels of H_2_O_2_ in Callisto treatment (Fig 3a) may be associated with the presence of mesotrione in synergy with the adjuvants and the decrease of this levels may be associated with the degradation of mesotrione and an efficient antioxidative system.

MDA is an indicator of lipid peroxidation, therefore a positive correlation between the MDA and H_2_O_2_ levels could indicate that the latter is a causative agent of oxidative stress [41, 42, 43]. Nevertheless, the two *B*. *megaterium* studied in this work exhibited opposing behaviors regarding MDA and H_2_O_2_ levels, mainly after treatment with the herbicides (Fig 3). While for *B*. *megaterium* CCT 7729 the MDA rates at lag phase were higher than mid log phase, the opposite condition for herbicides treatments was found.

Previous studies have shown that the *B*. *megaterium* isolate L1 and *Bacillus subtilis* isolate B19 can tolerate high levels of oxidative stress caused by the direct effect of xenobiotics and/or the intermediates generated during the biodegradation of these compounds [17, 43]. Free radicals and ROS such as H_2_O_2_ react with unsaturated fatty acids in the cell membrane, release toxic compounds including aldehydes, and cause cell damage [16].

The different correlations among MDA (Fig 3b) and H_2_O_2_ levels (Fig 3a), as the causative agent of lipid peroxidation, probably are due to a specific pattern of saturation of membrane lipids for each *B*. *megaterium* strain studied in this work. This pattern is dependent upon the presence of herbicides in the culture media and to adaptation strategies to the aquatic and terrestrial environments. The additional results of degradation and lipid saturation presented and discussed in following sections support this hypothesis.

### Degradation of mesotrione

One adaptive response to herbicides found in some tolerant micro-organisms is the ability to metabolize xenobiotics [25]. Previous studies showed the ability of some micro-organisms in metabolize/degrade mesotrione, as *E*. *coli* DH5-α [5], which can degrade mesotrione after 3 h of contact, *P*. *ananatis* CCT 7673 [4], after 18 h for, and *B*. *megaterium* 3B6 after 25 h [7]. Degradation products of mesotrione were also reported by Batisson et al. [3] for *Bacillus* sp. as 2-amino-4-methyl sulfonyl benzoic acid (AMBA) and 4-methylsulfonyl-2-nitrobenzoic acid (MNBA), which were more toxic than the original mesotrione molecule [2, 8, 40]. Pileggi et al. [4] identified different metabolites from MNBA and AMBA after mesotrione degradation by *P*. *ananatis*.

In the present study, HPLC-DAD and LC-ESI-MS/MS methods were applied to quantify the mesotrione on different incubation time and to evaluate the new compounds/metabolites that can appear in the degrading process, respectively. We raised the possibility of existing new routes of mesotrione degradation in the two *B*. *megaterium* strains, based on the fact of that the metabolites observed are not related with the ones described by Durand et al. [7], Batisson et al. [3], Pileggi et al. [4].

Different herbicide degradation pathways could be an interesting characteristic for bioremediation of contaminated areas, because toxic metabolites could be avoided and the *Bacillus* genus enhance this biotechnological use due to its high rates of degradation of different herbicides and mineralization ability of these compounds [44, 45, 46].

### Antioxidant enzymes

#### SOD

The activities of enzymes of the antioxidant defense system are generally increased in microorganisms under oxidative stress conditions to maintain a stable cellular environment and prevent damage [17, 47, 48]. One of these enzymes, SOD, belongs to a family composed of four types of metalloenzymes that are dependent on the metal cofactors Cu/Zn, Mn, Fe, or Ni [9]. These enzymes can detoxify oxygen radicals by conversion of O_2_^-•^ radical into H_2_O_2_ and O_2_.

Previous studies on Mn-SOD isoforms in *Burkholderia cepacia* and *Micrococcus* sp. demonstrated that its activity was modulated by oxidative stress and was associated with the herbicide detoxification processes [49, 50]. Mn-SOD, and Fe-SOD, are found in the cytoplasm and are regulated by iron levels [21].

The specific activity of Mn-SOD1 isoform was observed only in CCT 7729, at the log phase of growth in the control and mesotrione treatment. This isoform was probably inhibited by Callisto. The Mn-SOD2 isoform activity was present and with high activity in all times and treatments of CCT 7730, except for 3 h at MMC treatment. This increased activity may contribute to the increased production of H_2_O_2_, and MDA, mainly at log phase, for CCT 7730 (Fig 3). Although a densitometric analysis of the bands was not carried out, Mn-SOD2 accounts for almost all SOD activity in both isolates. Yet, the low, but induced Mn-SOD1 activity at 14 h in MM and MMM for CCT 7729, indicates distinct responses by the two isolates and in relation to the treatments applied.

High SOD activity is necessary to prevent damage and ensure cell growth because SOD is the only enzyme that can control the concentration of the two substrates via the Haber-Weiss reaction; these substrates produce the OH^•^ radical [51]. The presence of two SOD isoforms in CCT 7729 (Figs 4 and 5) might be responsible for the better growth rates in culture medium, but also represents an adaptation to oligotrophic environment. Furthermore, interferences caused by macro- and micro-nutrient limitations on SOD activity were shown for the microalga *Dunaliella salina* [52].

#### CAT

The CAT1 enzyme was found exclusively in the CCT 7729 strain, with the highest activity at log phase in the mesotrione and Callisto treatments. CAT2 was found in both strains, with the higher activity more restricted to the lag phase and in the CCT 7730 strain. The most interesting fact was that this isoform was not expressed in the Callisto treatments. The CAT3, which should correspond to HPII presents greater activities in CCT 7730 at lag phase. The most highlight datum is the absence of activity in log phase in the Callisto treatment in the CCT 7729 strain, maybe in an activity compensation manner by CAT1. Nevertheless, the CAT4 isoform, which may correspond to HPI, also shows a higher activity in CCT 7730 than CCT 7729, especially in the Callisto treatments, but in log phase. It is clearly accounting for the majority of CAT activity and H_2_O_2_ degradation, although other peroxidases not analyzed in this study may also be involved.

A similar pattern was found for SOD, with two isoforms in CCT 7729 strain and only one isoform and higher activity in CCT 7730 strain (Fig 5). A better control of the levels of H_2_O_2_ and MDA (Fig 3) could be reached by CTT 7729 through the SOD and CAT polymorphism. These responses led this strain to overcome the oxidative stress better than CTT 7730 (Fig 1).

Peroxidases, such as Ahp, are active when the CAT levels are low, whereas CAT is active when the H_2_O_2_ levels are high or when the cells lack nutrients [21]. The failure of isolate CCT7730 to adapt in the culture media (as observed by the lower growth rates compared with CCT 7729, Fig 1) may be associated with this state with a more intense CAT activity (Fig 6). Different studies have demonstrated the interference of CAT activity on the tolerance of bacteria to xenobiotics [9, 17, 23, 43].

In this context, *B*. *megaterium* CCT 7729 exhibited increased CAT activity in response to the herbicide, but was well distributed in 4 isoforms, with specific herbicide activities (CAT1). In contrast, isolate CCT 7730 needed to maintain a high CAT level even in the absence of the herbicide (Fig 6), possibly due to its lack of adaptation to the mineral medium. These data are probably related to adaptations to the water environment, in which the CCT 7729 strain was isolated, more restricted in nutrients than agricultural soil, in which the CCT 7730 strain was isolated.

### Lipid saturation

Changes in membrane fluidity are a primary effect observed in herbicide-tolerant bacteria and are caused by lipid peroxidation [12]. This adjustment in the composition and structure of membrane lipids can reduce the damage caused by ROS, decrease the energy expenditure, limit the entry of xenobiotics into the cell and optimize cell growth [5, 53, 54].

FTIR and PCA analysis showed differences in the saturation patterns in the lag and log phases after mesotrione and Callisto treatments for both isolates (Fig 7, S4 and S5 Figs, S1 Table). Olchanheski et al. [5] reported that mesotrione induced changes in the saturation of membrane lipids in *E*. *coli* DH5-α. The membrane fluidity of another strain of *E*. *coli* decreased after exposure to the herbicide 2,4-dichlorophenoxyacetic acid, which prevented the entry of xenobiotics into the bacterial cell and thereby allowed the bacteria to withstand chemical damage [53]. In *E*. *coli*, the composition of membrane lipids changes during growth in the presence of ethanol while the area available for the passive process of ion diffusion decreases [55].

The adjuvants present in Callisto are comprised of alcoholic groups (1,2-benzisothiazolin-3-one, 1-octanol, and poly (oxy-1,2-ethanediyl), alpha-isodecyl-omega-hydroxy-phosphate). The compound 1-octanol was shown to modify the membrane lipids in *Pseudomonas oleovorans* [56]. Therefore, these adjuvants may contribute to the lipid changes observed in the treatments with MMC in both bacterial isolates compared with treatment with MM (S4 and S5 Figs, S1 Table).

MDA is an aldehyde produced during reactions with ROS (particularly H_2_O_2_) in the presence of unsaturated fatty acids in the cell membrane. Therefore, higher rates of membrane lipid unsaturation may be associated with high rates of MDA. Accordingly, the changes observed in the lipid saturation levels (Fig 7, S4 and S5 Figs, S1 Table) are most likely associated with changes in membrane fluidity as a defense strategy against herbicide entry and the degradation metabolites. In this respect, the MDA levels observed (Fig 3b) should not only represent the amount of H_2_O_2_ produced (Fig 3a) but also the amount of H_2_O_2_ in relation to the unsaturated lipids available for peroxidation; the amount of the latter depends on the level of induction by herbicides (Fig 7, S4 and S5 Figs, S1 Table).

### System responses to herbicides

**Fig 8:**
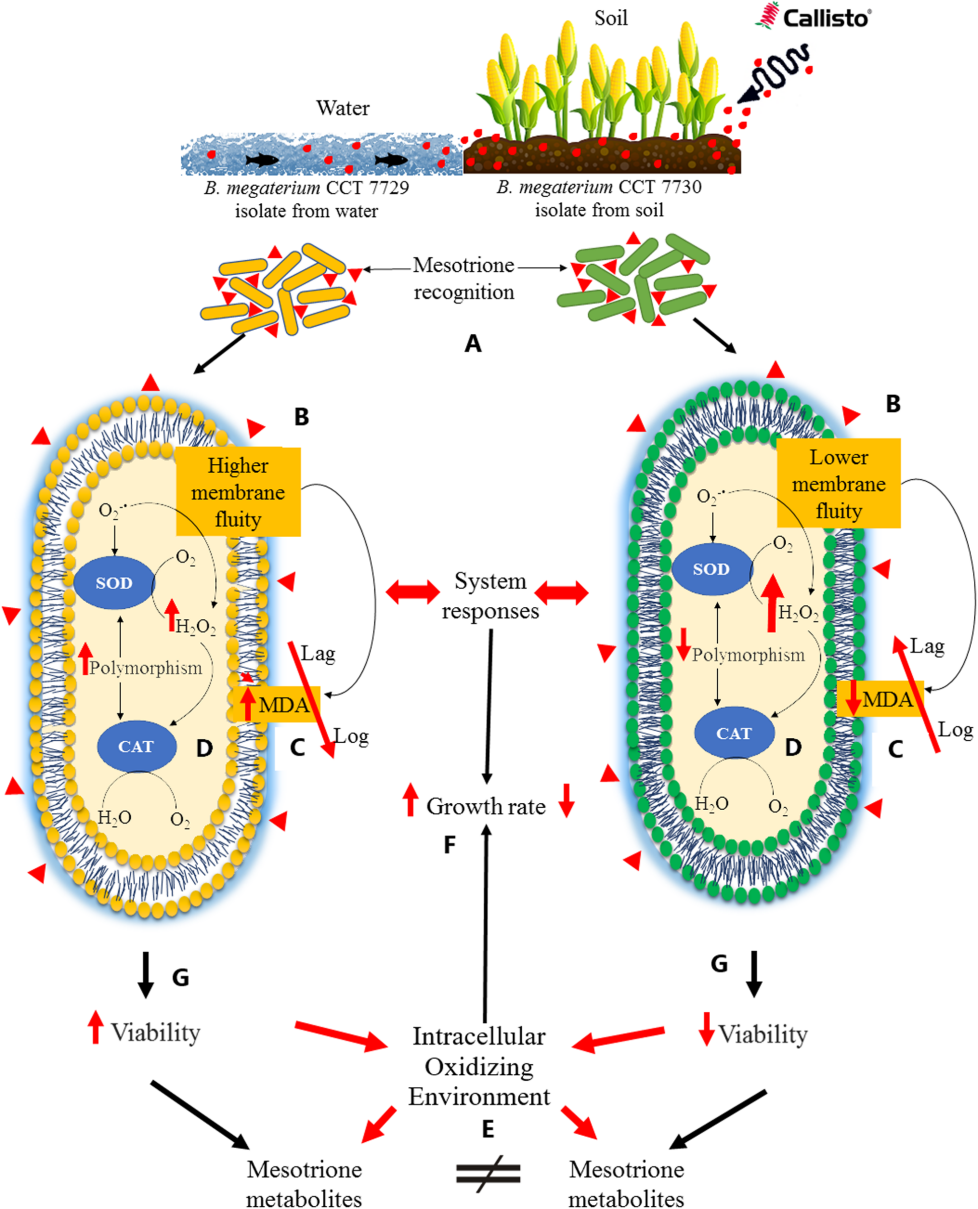
Graphical representation of the main results showing the different responses system to herbicides of *B*. *megaterium* CCT 7729 and CCT 7730. These strains were originated from water and soil environments contaminated with Callisto (A), respectively. The response system starts with different lipid saturation levels (B), forming a cascade characterized by different levels of membrane permeability and MDA rates (C), interfering with herbicide entry into the bacterial cells and inducing different antioxidant responses (D). Therefore, different levels of H_2_O_2_ between the two strains were created, originating distinct intracellular oxidizing environments (E) for each strain and, therefore, different degradation routes. These response systems interfered with the growth ability (F) and viability rates (G) and may be associated with the nutrient availability of the isolation environments of each strain (A). ↑ Represents significant increase compared with the control and ↓ represents significant decrease compared with the control.

The FTIR and PCA analysis revealed that the pattern of lipid saturation was modified in different ways between the isolates, and in the presence of mesotrione active ingredient and Callisto (Figs 7 and 8), indicating that membrane permeability was changed, higher in CCT 7729 strain, to protect the cells against herbicide-induced stress in strain-dependent strategies. Therefore, herbicide uptake rates should differ for each of the strains studied in this report.

The final balance of ROS production differed in the two isolates (Fig 3). H_2_O_2_ exhibits a very extensive network of interactions in the cellular metabolism [57], and influence the permeability of membranes [58]. H_2_O_2_ may also interfere with cellular metabolism by inducing alternative biochemical routes [59], especially when promiscuous enzymes are involved in the degradation process [60, 61, 62]. H_2_O_2_ may have played an important role in the differential responses to herbicides in strains CCT 7729 and CCT 7730, likely interfering with SOD and CAT isoforms activities (Figs 5 and 6) [63] and consequently with growth and viability rates (Figs 1 and 2).

The analysis of the degradation metabolites of mesotrione in the *B*. *megaterium* isolates (S1, S2 and S3 Figs) indicated the presence of two pathways: one in which the nitro functional group is maintained (in isolate CCT 7729) and another in which it is lost (in isolate CCT 7730).

The different responses of these isolates to herbicides, including the different lipid saturation levels (Fig 7, S4 and S5 Figs, S1 Table), probably formed a system of cascade responses characterized by different levels of membrane permeability and MDA rates (Fig 3b), interfering with herbicide entry into the bacterial cells and inducing different antioxidant responses (Figs 5 and 6). Therefore, different levels of H_2_O_2_ between the two *B*. *megaterium* strains were created (Fig 3a), originating from distinct intracellular oxidizing environments for each strain, and therefore different degradation routes (S1, S2 and S3 Figs). This hypothesis is consistent with the theory of enzyme promiscuity in which enzymes may be involved in unusual metabolic pathways under different conditions [60, 61]. Our findings and hypotheses can be summarized in Fig 8.

## Conclusions

Two strains of *B*. *megaterium* originating from water and soil environments contaminated with Callisto were studied with respect to their cellular and enzymatic responses to the stress produced by this herbicide. The cell viability, the peroxide and MDA levels corroborated the hypothesis of the higher toxicity of Callisto due to the presence of adjuvants. However, the response system of each isolate was distinct. The changes in the pattern of lipid saturation were different, indicating that the membrane permeability might have been changed in time and in presence of herbicides. Therefore, it is possible that the herbicide was absorbed at different rates by these isolates during the degradation process and induced distinct redox conditions in the cell. Accordingly, the changes observed in the lipid saturation levels were probably associated with changes in membrane fluidity as a defense strategy against herbicide entry. This differential input of xenobiotic substance led to different SOD and CAT activities and isoforms expressed in response to the herbicides. For this reason, the MDA levels observed should not only represent the amount of H_2_O_2_ produced but also the amount of H_2_O_2_ in comparison to the unsaturated lipids available for peroxidation. Therefore, the final balance of ROS produced was different between the two isolates and may have interfered with different metabolic pathways, not described yet, and thus the degradation ability of these isolates. Furthermore, these characteristic response systems interfered with the growth ability and viability rates and may be associated with the nutrient richness of the original isolation environments of each strain. These effects of redox environments in agricultural soils on metabolic pathways should be considered when evaluating the impact of herbicides, particularly in water ecosystems where different levels of metabolites toxicity may occur. The transport of herbicides from agricultural soil to aquatic environments is a common fate for these xenobiotics compounds, and may interfere in distinct ways on survival and degradation rates, even on phylogenetically similar bacteria. This, in turn, has consequences to soil and water microbiota and environmental contamination levels.

## Supporting information

S1 File Growth rate. Statistical analyzes.
(PDF)

S2 File Viability. Statistical analyzes.
(PDF)

S3 File Peroxide. Statistical analyzes.
(PDF)

S4 File MDA. Statistical analyzes.
(PDF)

S1 Fig Spectrogram 1. A- LC-(+)ESIMS/MS analysis for mesotrione (R_t_ = 22.9 min.) added in the media at 24 h of incubation A- *B*. *megaterium* CCT 7729. B- *B*. *megaterium* CCT 7730.
(PDF)

S2 Fig Spectrogram 2. A- LC-(+)ESIMS/MS analysis for mesotrione (R_t_ = 22.9 min.) added in the media at 0 h of incubation of *B*. *megaterium* CCT 7729. B- Extract peak chromatogram in R_t_ = 22.9 min. C- MS spectrum of peak in R_t_ = 22.9 min. D- LC-MS/MS spectrum of ion of *m/z* 340 [M+H]^+^.
(PDF)

S3 Fig Spectrogram 3. A- LC-(+)ESIMS/MS analysis for mesotrione (R_t_ = 22.9 min) added in the media at 24 h of incubation of *B*. *megaterium* CCT 7729. B- Extract peak chromatogram in R_t_ = 17.8 min. C- MS spectrum of peak in R_t_ = 17.8 min.
(PDF)

S4 Fig PCA analysis. PCA of the structure of lipid saturation of *B*. *megaterium* CCT 7729 (white spots) and *B*. *megaterium* CCT 7730 (dark spots) based on one million variables. PCA groups the data according to the degree of similarity. Samples with greater similarity are grouped in the same quadrant.
(PDF)

S5 Fig Regression model. Regression model of vector FTIR spectrum extract in strains: (a) CCT 7729 and (b) CCT 7730.
(PDF)

S1 Table Quantitative analysis of infrared spectra. Quantitative analysis of infrared spectra of bands corresponding to alkanes C-H_3_ stretching (2956 and 2872 cm^-1^) and C-H_2_ bonds (2920 and 2850 cm^-1^); C = O stretching (1,750 cm^-1^); long chain hydrocarbons containing (-CH_2_-)n (1477–1452 cm^-1^ and 725 cm^-1^); -CH_3_ group indicative of the isopropyl group (1380 to 1350 cm^-1^); C-O group stretching vibration region of esters (1229 cm^-1^). Treatments (T column): 1–6—*Bacillus megaterium* CCT 7729; 7–12—*Bacillus megaterium* CCT 7730; 1–3 and 7–9–3 h of incubation; 4–6 and 10–12–14 h of incubation; 1, 4, 7 and 10 –MM; 2, 5, 8 and 11 –MMM; 3, 6, 9 and 12 –MMC.
(PDF)
