# Supplementary material for: Bacillus megaterium strains derived from water and soil exhibit differential responses to the herbicide mesotrione
Source: PLoS One. 2018 Apr 25;13(4):e0196166. doi: 10.1371/journal.pone.0196166 (PMC5918998; doi:10.1371/journal.pone.0196166)
Supplement: S1 File — Statistical analyzes. (PDF) [file pone.0196166.s001.pdf]

S1 File. Growth rate.

Significant differences are indicated in red.

Sect 1.1

Growth rate; *B. megaterium* CCT 7729; end of log phase (22h); bonferroni means st

	Summary of Growth rate	
	B. megaterium CCT 7729	
	Mean	Std. Dev.
MM	5.2289999	.0890954
MMM	5.0429999	.13777139
MMC	4.7640001	.05992504
Total	4.984875	.21671854

Analysis of Variance

Source	SS	df	MS	F	Prob > F
Between groups	.275686568	2	.137843284	12.98	0.0105
Within groups	.053081921	5	.010616384		
Total	.328768489	7	.046966927		

Comparison of Growth rate by *B. megaterium* CCT 7729; 22h

(Bonferroni)

Row Mean-Col Mean	MM	MMM
MMM	-.186	
	0.315	
MMC	-.465	-.279
	0.013	0.063

Section 1.2

Growth rate; *B. megaterium* CCT 7730; end of log phase (22h); bonferroni means st

	Summary of Growth rate *B. megaterium* CCT 7730 Mean	Std. Dev.
MM	1.069	.0445309
MMM	1.335	.07607235
MMC	.78866667	.09246258
Total	1.0642222	.24506673

Analysis of Variance

Source	SS	df	MS	F	Prob > F
Between groups	.447822953	2	.223911476	41.16	0.0003
Within groups	.032638665	6	.005439777		
Total	.480461618	8	.060057702		

Comparison of Growth rate by *B. megaterium* CCT 7730; 22h

(Bonferroni)

Row Mean- Col Mean	MM	MMM
MMM	.266	
	0.013	
MMC	-.280333	-.546333
	0.010	0.000

Section 1.3

Growth rate; *B. megaterium* CCT 7729 (L1) x *B. megaterium* CCT 7730 (L2); end of log phase (22h); bonferroni means st

Bartlett's test for equal variances: chi2(5) = 2.3610 Prob>chi2 = 0.797

	Summary of Growth rate	
	B. megaterium CCT 7729 x *B. megaterium* CCT 7730; 22h	
	Mean	Std. Dev.
L1 MM	5.2289999	.0890954
L1 MMM	5.0429999	.13777139
L1 MMC	4.7640001	.05992504
L2 MM	1.069	.0445309
L2 MMM	1.335	.07607235
L2 MMC	.78866667	.09246258
Total	2.9092353	2.0296571

Analysis of Variance

Source	SS	df	MS	F	Prob > F
Between groups	65.8264085	5	13.1652817	1689.42	0.0000
Within groups	.085720586	11	.007792781		
Total	65.9121291	16	4.11950807		

Comparison of Growth rate by *B. megaterium* CCT 7729 x *B. megaterium* CCT 7730; 22h

(Bonferroni)

Row Mean- / Col Mean	L1 MM	L1 MMM	L1 MMC	L2 MM	L2 MMM
L1 MMM	-.186				
	0.622				
L1 MMC	-.465	-.279			
	0.002	0.039			
L2 MM	-4.16	-3.974	-3.695		
	0.000	0.000	0.000		
L2 MMM	-3.894	-3.708	-3.429	.266	
	0.000	0.000	0.000	0.053	
L2 MMC	-4.44033	-4.25433	-3.97533	-.280333	-.546333
	0.000	0.000	0.000	0.038	0.000
