# Supplementary material for: Bacillus megaterium strains derived from water and soil exhibit differential responses to the herbicide mesotrione
Source: PLoS One. 2018 Apr 25;13(4):e0196166. doi: 10.1371/journal.pone.0196166 (PMC5918998; doi:10.1371/journal.pone.0196166)
Supplement: S2 File — Statistical analyzes. (PDF) [file pone.0196166.s002.pdf]

**S2 File. Viability.**

**Section 2.1**

Viability *B. megaterium* CCT 7729 (L1); MM, MMM, and MMC; 3 and 14h; bonferroni means st

Bartlett's test for equal variances: chi2(5) = 9.4599 Prob>chi2 = 0.092

| L1 Treatment | Summary of Viability (LOG de UFC mL -1) Mean | Std. Dev. |
|---|---|---|
| MM 3 | 149.915 | 10.486395 |
| MM 14 | 250 | 36.062446 |
| MMM 3 | 125.89 | 1.4547516 |
| MMM 14 | 55.222221 | 33.533288 |
| MMC3 | 176.25 | 7.4246212 |
| MMC 14 | 299.33334 | 19.79899 |
| Total | 163.88095 | 85.971661 |

Analysis of Variance

| Source | SS | df | MS | F | Prob > F |
|---|---|---|---|---|---|
| Between groups | 91973.8605 | 5 | 18394.7721 | 35.80 | 0.0000 |
| Within groups | 4110.78485 | 8 | 513.848106 | | |
| Total | 96084.6454 | 13 | 7391.12657 | | |

Comparison of Viability (LOG de UFC mL -1) by *B. megaterium* CCT 7729 (L1); 3 and 14h Bonferroni)

| Row Mean-Col Mean | MM3 | MM14 | MMM3 | MMM14 | MMC3 |
|---|---|---|---|---|---|
| MM14 | 100.085 | | | | |
| | 0.034 | | | | |
| MMM3 | -24.025 | -124.11 | | | |
| | 1.000 | 0.005 | | | |
| MMM14 | -94.6928 | -194.778 | -70.6678 | | |
| | 0.027 | 0.000 | 0.077 | | |
| MMC3 | 26.335 | -73.75 | 50.36 | 121.028 | |
| | 1.000 | 0.175 | 0.615 | 0.006 | |
| MMC14 | 149.418 | 49.3333 | 173.443 | 244.111 | 123.083 |
| | 0.003 | 0.918 | 0.000 | 0.000 | 0.009 |

**Section 2.2**

Viability, by *B. megaterium* CCT 7730 (L2); MM, MMM AND MMC, 3 and 14h

Bartlett's test for equal variances: chi2(5) = 20.9112 Prob>chi2 = 0.001

Viability, by *B. megaterium* CCT 7730 (L2); MM, MMM and MMC; 3 and 14h

Test: Equality of populations (Kruskal-Wallis test)

| L2 | _Obs | _RankSum |
|---|---|---|
| MM3 | 2 | 11.00 |
| MM14 | 2 | 25.00 |
| MMM3 | 2 | 15.00 |
| MMM14 | 2 | 20.00 |
| MMC3 | 3 | 9.00 |
| MMC14 | 2 | 11.00 |

chi-squared = 8.967 with 5 d.f.

probability = 0.1104

chi-squared with ties = 8.967 with 5 d.f.

probability = 0.1104

**Section 2.3**

Viability *B. megaterium* CCT 7729 (L1); MM3 X *B. megaterium* CCT 7730 (L2); MM3, MM14 and MMM3, bonferroni means st

Bartlett's test for equal variances: chi2(3) = 5.8072 Prob>chi2 = 0.121

| L1 x L2 Treatments | Summary of Viability (LOG de UFC mL -1) Mean | Std. Dev. |
|---|---|---|
| L1 MM3 | 149.915 | 10.486395 |
| L2 MM3 | 83.833332 | 4.4783447 |
| L2 MM14 | 239.25 | .35355339 |
| L2 MMM3 | 127.25 | 1.0606602 |
| Total | 150.06208 | 60.773067 |

Analysis of Variance

| Source | SS | df | MS | F | Prob > F |
|---|---|---|---|---|---|
| Between groups | 25722.2895 | 3 | 8574.0965 | 261.27 | 0.0000 |
| Within groups | 131.270049 | 4 | 32.8175122 | | |
| Total | 25853.5596 | 7 | 3693.36565 | | |

Comparison of Viability (LOG de UFC mL -1) by *B. megaterium* CCT 7729 (L1); MM3 x *B. megaterium* CCT 7730 (L2); MM3, MM14 and MMM3

(Bonferroni)

| Row Mean-Col Mean | L1 MM3 | L2 MM3 | L2 MM14 |
|---|---|---|---|
| L2 MM3 | -66.0817 | | |
| | 0.002 | | |
| L2 MM14 | 89.335 | 155.417 | |
| | 0.001 | 0.000 | |
| L2 MMM3 | -22.665 | 43.4167 | -112 |
| | 0.100 | 0.010 | 0.000 |

**Section 2.4**

Viability; *B. megaterium* CCT 7729 (L1); MM3 X *B. megaterium* CCT 7730 (L2); MMM14, MMC3 and MMC15, bonferroni means st

Bartlett's test for equal variances: chi2(3) = 9.8573 Prob>chi2 = 0.020

Test: Equality of populations (Kruskal-Wallis test)

| L1 X L2 | _Obs | _RankSum |
|---|---|---|
| L1 MM3 | 2 | 17.00 |
| L2 MMM14 | 2 | 12.00 |
| L2 MMC3 | 3 | 9.00 |
| L2 MMC14 | 2 | 7.00 |

chi-squared = 5.733 with 3 d.f.

probability = 0.1253

chi-squared with ties = 5.733 with 3 d.f.

probability = 0.1253

**Section 2.5**

Viability; *B. megaterium* CCT 7729 (L1); MM14 X *B. megaterium* CCT 7730 (L2); MM3, MM14 and MMM3, bonferroni means st

Bartlett's test for equal variances: chi2(3) = 10.5843 Prob>chi2 = 0.014

Test: Equality of populations (Kruskal-Wallis test)

| L1 X L2 | _Obs | _RankSum |
|---|---|---|
| L1 MM14 | 2 | 13.00 |
| L2 MM3 | 2 | 3.00 |
| L2 MM14 | 2 | 13.00 |
| L2 MMM3 | 2 | 7.00 |

chi-squared = 6.000 with 3 d.f.

probability = 0.1116

chi-squared with ties = 6.000 with 3 d.f.

probability = 0.1116

**Section 2.6**

Viability; *B. megaterium* CCT 7729 (L1); MM14 x *B. megaterium* CCT 7730 (L2); MMM14, MMC3 and MMC14, bonferroni means st

Bartlett's test for equal variances: chi2(3) = 8.8615 Prob>chi2 = 0.031

```
Test: Equality of populations (Kruskal-Wallis test)

L1 x L2          _Obs   _RankSum
L1 MM14             2      17.00
L2 MMM14            2      12.00
L2 MMC3             3       9.00
L2 MMC14            2       7.00

chi-squared =     5.733 with 3 d.f.
probability =     0.1253

chi-squared with ties =     5.733 with 3 d.f.
probability =     0.1253
```

**Section 2.7**

Viability *B. megaterium* CCT 7729 (L1); MMM3 X *B. megaterium* CCT 7730 (L2); MM3, MM14 and MMM3, bonferroni means st

Bartlett's test for equal variances: chi2(3) = 4.1104 Prob>chi2 = 0.250

| L1 X L2 | Summary of Viability (LOG de UFC mL -1) Mean | Std. Dev. |
|---|---|---|
| L1 MMM3 | 125.89 | 1.4547516 |
| L2 MM3 | 83.833332 | 4.4783447 |
| L2 MM14 | 239.25 | .35355339 |
| L2 MMM3 | 127.25 | 1.0606602 |
| Total | 142.03741 | 58.009799 |

Analysis of Variance

| Source | SS | df | MS | F | Prob > F |
|---|---|---|---|---|---|
| Between groups | 26895.5563 | 3 | 8965.18543 | 1755.25 | 0.0000 |
| Within groups | 25.5381761 | 5 | 5.10763523 | | |
| Total | 26921.0945 | 8 | 3365.13681 | | |

Comparison of Viability (LOG de UFC mL -1) by *B. megaterium* CCT 7729 (L1); MMM3 X *B. megaterium* CCT 7730 (L2); MM3, MM14 and MMM3

(Bonferroni)

```
Row Mean-|
Col Mean |    L1 MMM3     L2 MM3      L2 MM14
---------+---------------------------------
  L2 MM3 |   -42.0567
         |      0.000
         |
 L2 MM14 |     113.36    155.417
         |      0.000      0.000
         |
 L2 MMM3 |       1.36    43.4167        -112
         |      1.000      0.000       0.000
```

**Section 2.8**

Viability; *B. megaterium* CCT 7729 (L1); MMM3 X *B. megaterium* CCT 7730 (L2); MMM14, MMC3 and MMC14, bonferroni means st

Bartlett's test for equal variances: chi2(3) = 16.9681 Prob>chi2 = 0.001

Test: Equality of populations (Kruskal-Wallis test)

| L1 X L2 | _Obs | _RankSum |
|---|---|---|
| L1 MMM3 | 3 | 18.00 |
| L2 MMM14 | 2 | 18.00 |
| L2 MMC3 | 3 | 9.00 |
| L2 MMC14 | 2 | 10.00 |

chi-squared = 4.855 with 3 d.f.

probability = 0.1828

chi-squared with ties = 4.855 with 3 d.f.

probability = 0.1828

**Section 2.9**

Viability, *B. megaterium* CCT 7729 (L1); MM14 X *B. megaterium* CCT 7730 (L2); MM3, MM14 and MMM3, bonferroni means st

Bartlett's test for equal variances: chi2(3) = 11.3444 Prob>chi2 = 0.010

Test: Equality of populations (Kruskal-Wallis test)

| L1 X L2 | _Obs | _RankSum |
|---|---|---|
| L1 MMM14 | 3 | 8.00 |
| L2 MM3 | 2 | 7.00 |
| L2 MM14 | 2 | 17.00 |
| L2 MMM3 | 2 | 13.00 |

chi-squared = 6.644 with 3 d.f.

probability = 0.0841

chi-squared with ties = 6.644 with 3 d.f.

probability = 0.0841

**Section 2.10**

Viability; *B. megaterium* CCT 7729 (L1); MMM14 X *B. megaterium* CCT 7730 (L2) MMM14, MMC3 and MMC14, bonferroni means st

Bartlett's test for equal variances: chi2(3) = 9.2083 Prob>chi2 = 0.027

Viability, by *B. megaterium* CCT 7729 (L1); MMM14 X *B. megaterium* CCT 7730 (L2) MMM14, MMC3 and MMC14

Test: Equality of populations (Kruskal-Wallis test)

| L1 X L2 | _Obs | _RankSum |
|---|---|---|
| L1 MMM14 | 3 | 11.00 |
| L2 MMM14 | 2 | 18.00 |
| L2 MMC3 | 3 | 15.00 |
| L2 MMC14 | 2 | 11.00 |

chi-squared = 3.855 with 3 d.f.

probability = 0.2776

chi-squared with ties = 3.855 with 3 d.f.

probability = 0.2776

**Section 2.11**

Viability; *B. megaterium* CCT 7729 (L1); MMC3 X *B. megaterium* CCT 7730 (L2) MM3, MM14 and MMM3, bonferroni means st

Bartlett's test for equal variances: chi2(3) = 4.7675 Prob>chi2 = 0.190

| L1 X L2 | Summary of Viability (LOG de UFC mL -1) Mean | Std. Dev. |
|---|---|---|
| L1 MMC3 | 176.25 | 7.4246212 |
| L2 MM3 | 83.833332 | 4.4783447 |
| L2 MM14 | 239.25 | .35355339 |
| L2 MMM3 | 127.25 | 1.0606602 |
| Total | 156.64583 | 61.902602 |

Analysis of Variance

| Source | SS | df | MS | F | Prob > F |
|---|---|---|---|---|---|
| Between groups | 26747.0941 | 3 | 8915.69804 | 466.60 | 0.0000 |
| Within groups | 76.4305717 | 4 | 19.1076429 | | |
| Total | 26823.5247 | 7 | 3831.9321 | | |

Comparison of Viability (LOG de UFC mL -1) by *B. megaterium* CCT 7729 (L1); MMC3 X *B. megaterium* CCT 7730 (L2) MM3, MM14 and MMM3

(Bonferroni)

| Row Mean- Col Mean | L1 MMC3 | L2 MM3 | L2 MM14 |
|---|---|---|---|
| L2 MM3 | -92.4167 | | |
| | 0.000 | | |
| L2 MM14 | 63 | 155.417 | |
| | 0.001 | 0.000 | |
| L2 MMM3 | -49 | 43.4167 | -112 |
| | 0.002 | 0.003 | 0.000 |

**Section 2.12**

Viability; *B. megaterium* CCT 7729 (L1); MMC3 X *B. megaterium* CCT 7730 (L2) MMM14, MMC3 and MMC14, bonferroni means st

Bartlett's test for equal variances: chi2(3) = 10.3204 Prob>chi2 = 0.016

Viability, by *B. megaterium* CCT 7729 (L1); MMC3 X *B. megaterium* CCT 7730 (L2) MMM14, MMC3 and MMC14

Test: Equality of populations (Kruskal-Wallis test)

| L1 X L2 | _Obs | _RankSum |
|---|---|---|
| L1 MMC3 | 2 | 17.00 |
| L2 MMM14 | 2 | 12.00 |
| L2 MMC3 | 3 | 9.00 |
| L2 MMC14 | 2 | 7.00 |

chi-squared = 5.733 with 3 d.f.

probability = 0.1253

chi-squared with ties = 5.733 with 3 d.f.

probability = 0.1253

**Section 2.13**

Viability; *B. megaterium* CCT 7729 (L1); MMC14 X *B. megaterium* CCT 7730 (L2) MM3, MM14 and MMM3; bonferroni means st

Bartlett's test for equal variances: chi2(3) = 8.1483 Prob>chi2 = 0.043

Viability, by *B. megaterium* CCT 7729 (L1); MMC14 X *B. megaterium* CCT 7730 (L2) MM3, MM14 and MMM3

Test: Equality of populations (Kruskal-Wallis test)

| L1 X L2 | _Obs | _RankSum |
|---|---|---|
| L1 MMC14 | 2 | 15.00 |
| L2 MM3 | 2 | 3.00 |
| L2 MM14 | 2 | 11.00 |
| L2 MMM3 | 2 | 7.00 |

chi-squared = 6.667 with 3 d.f.

probability = 0.0833

chi-squared with ties = 6.667 with 3 d.f.

probability = 0.0833

**Section 2.14**

Viability; *B. megaterium* CCT 7729 (L1); MMC14 X *B. megaterium* CCT 7730 (L2) MMM14, MMC3 and MMC14; bonferroni means st

Bartlett's test for equal variances: chi2(3) = 9.1364 Prob>chi2 = 0.028

Viability, by *B. megaterium* CCT 7729 (L1); MMC14 X *B. megaterium* CCT 7730 (L2) MMM14, MMC3 and MMC14

```
Test: Equality of populations (Kruskal-Wallis test)


  L1 X L2          _Obs   _RankSum

L1 MMC14             2      17.00

L2 MMM14             2      12.00

 L2 MMC3             3       9.00

L2 MMC14             2       7.00


chi-squared =     5.733 with 3 d.f.

probability =     0.1253


chi-squared with ties =     5.733 with 3 d.f.

probability =     0.1253
```
