# Supplementary material for: Bacillus megaterium strains derived from water and soil exhibit differential responses to the herbicide mesotrione
Source: PLoS One. 2018 Apr 25;13(4):e0196166. doi: 10.1371/journal.pone.0196166 (PMC5918998; doi:10.1371/journal.pone.0196166)
Supplement: S3 File — Statistical analyzes. (PDF) [file pone.0196166.s003.pdf]

S3 File. Peroxide.

Section 3.1

Peroxide, *B. megaterium* CCT 7729 (L1); MM, MMM and MMC; 3 and 14h; bonferroni means st

Bartlett's test for equal variances: chi2(5) = 3.6281 Prob>chi2 = 0.604

L1	Summary of peroxide ((mMol de H2O2/106 U.F.C.) Mean	Std. Dev.
L1 MM3	.043662	.02058246
L1 MM14	1.324414	.10291232
L1 MMM3	.69859198	.08232986
L1 MMM14	.28622867	.0343041
L1 MMC3	3.871364	.04116483
L1 MMC14	1.193428	.01372167
Total	1.2362815	1.319624

Analysis of Variance

Source	SS	df	MS	F	Prob > F
Between groups	19.1346304	5	3.82692608	1101.15	0.0000
Within groups	.020852388	6	.003475398		
Total	19.1554828	11	1.74140753		

Comparison of peroxide ((mMol de H2O2/106 U.F.C.) by *B. megaterium* CCT 7729 (L1); MM, MMM and MMC, 3 and 14h (Bonferroni)

Row Mean-Col Mean	L1 MM3	L1 MM14	L1 MMM3	L1 MMM14	L1 MMC3
L1 MM14	1.28075				
	0.000				
L1 MMM3	.65493	-.625822			
	0.000	0.001			
L1 MMM14	.242567	-1.03819	-.412363		
	0.094	0.000	0.006		
L1 MMC3	3.8277	2.54695	3.17277	3.58514	
	0.000	0.000	0.000	0.000	
L1 MMC14	1.14977	-.130986	.494836	.907199	-2.67794
	0.000	1.000	0.002	0.000	0.000

Section 3.2

Peroxide; *B. megaterium* CCT 7730 (L2);_MM, MMM and MMC; 3 and 14h; bonferroni means st

Bartlett's test for equal variances: chi2(5) = 1.8165 Prob>chi2 = 0.874

L2	Summary of peroxide ((mMol de H2O2/106 U.F.C.) Mean	Std. Dev.
L2 MM3	.33474199	.02058248
L2 MM14	1.4133551	.09015305
L2 MMM3	.98967203	.08232986
L2 MMM14	1.3001574	.05488658
L2 MMC3	5.8216	.08232999
L2 MMC14	1.484508	.04116491
Total	1.8539557	1.8050414

Analysis of Variance

Source	SS	df	MS	F	Prob > F
Between groups	39.0631501	5	7.81263002	1565.11	0.0000
Within groups	.034942303	7	.004991758		
Total	39.0980924	12	3.25817437		

Comparison of peroxide ((mMol de H2O2/106 U.F.C.) by *B. megaterium* CCT 7730 (L2); MM, MMM and MMC 3 and 14h (Bonferroni)

Row Mean- Col Mean	L2 MM3	L2 MM14	L2 MMM3	L2 MMM14	L2 MMC3
L2 MM14	1.07861				
	0.000				
L2 MMM3	.65493	-.423683			
	0.001	0.005			
L2 MMM14	.965415	-.113198	.310485		
	0.000	1.000	0.048		
L2 MMC3	5.48686	4.40824	4.83193	4.52144	
	0.000	0.000	0.000	0.000	
L2 MMC14	1.14977	.071153	.494836	.184351	-4.33709
	0.000	1.000	0.003	0.524	0.000

Section 3.3

Peroxide; *B. megaterium* CCT 7729 (L1); MM3 X *B. megaterium* CCT 7730 (L2); MM3, MM14, MMM3; bonferroni means st

Bartlett's test for equal variances: chi2(3) = 2.5110 Prob>chi2 = 0.473

L1 x L2	Summary of peroxide ((mMol de H2O2/106 U.F.C.) Mean	Std. Dev.
L1 MM3	.043662	.02058246
L2 MM3	.33474199	.02058248
L2 MM14	1.4133551	.09015305
L2 MMM3	.98967203	.08232986
Total	.77513527	.59117522

Analysis of Variance

Source	SS	df	MS	F	Prob > F
Between groups	2.77202451	3	.924008169	193.46	0.0000
Within groups	.023880627	5	.004776125		
Total	2.79590513	8	.349488142		

Comparison of peroxide ((mMol de H2O2/106 U.F.C.)

by *B. megaterium* CCT 7729 (L1); MM3 X *B. megaterium* CCT 7730 (L2); MM3, MM14, MMM3;

(Bonferroni)

Row Mean-<br>Col Mean	L1 MM3	L2 MM3	L2 MM14
L2 MM3	.29108		
	0.050		
L2 MM14	1.36969	1.07861	
	0.000	0.000	
L2 MMM3	.94601	.65493	-.423683
	0.000	0.001	0.007

Section 3.4

Peroxide; *B. megaterium* CCT 7729 (L1); MM3 X *B. megaterium* CCT 7730 (L2); MMM14, MMC3 and MMC14; bonferroni means st

Bartlett's test for equal variances: chi2(3) = 1.1851 Prob>chi2 = 0.757

L1 x L2	Summary of peroxide ((mMol de H2O2/106 U.F.C.) Mean	Std. Dev.
L1 MM3	.043662	.02058246
L2 MMM14	1.3001574	.05488658
L2 MMC3	5.8216	.08232999
L2 MMC14	1.484508	.04116491
Total	2.1624818	2.3353036

Analysis of Variance

Source	SS	df	MS	F	Prob > F
Between groups	38.16359	3	12.7211967	4272.82	0.0000
Within groups	.011908951	4	.002977238		
Total	38.1754989	7	5.4536427		

Comparison of peroxide ((mMol de H2O2/106 U.F.C.)

by *B. megaterium* CCT 7729 (L1); MM3 X *B. megaterium* CCT 7730 (L2); MMM14, MMC3 and MMC14

(Bonferroni)

Row Mean-Col Mean	L1 MM3	L2 MMM14	L2 MMC3
L2 MMM14	1.2565		
	0.000		
L2 MMC3	5.77794	4.52144	
	0.000	0.000	
L2 MMC14	1.44085	.184351	-4.33709
	0.000	0.167	0.000

Section 3.5

Peroxide; *B. megaterium* CCT 7729 (L1); MM14 X *B. megaterium* CCT 7730 (L2); MM3, MM14 and MMM 3; bonferroni means st

Bartlett's test for equal variances: chi2(3) = 1.4534 Prob>chi2 = 0.693

L1 X L2	Summary of peroxide ((mMol de H2O2/106 U.F.C.) Mean	Std. Dev.
L1 MM14	1.324414	.10291232
L2 MM3	.33474199	.02058248
L2 MM14	1.4133551	.09015305
L2 MMM3	.98967203	.08232986
Total	1.0597468	.44865727

Analysis of Variance

Source	SS	df	MS	F	Prob > F
Between groups	1.57629886	3	.525432955	77.16	0.0001
Within groups	.034047935	5	.006809587		
Total	1.6103468	8	.20129335		

Comparison of peroxide ((mMol de H2O2/106 U.F.C.) by *B. megaterium* CCT 7729 (L1); MM14 X *B. megaterium* CCT 7730 (L2); MM3, MM14 and MMM 3

(Bonferroni)

Row Mean-Col Mean	L1 MM14	L2 MM3	L2 MM14
L2 MM3	-.989672		
	0.000		
L2 MM14	.088941	1.07861	
	1.000	0.000	
L2 MMM3	-.334742	.65493	-.423683
	0.059	0.003	0.015

Section 3.6

Peroxide; *B. megaterium* CCT 7729 (L1); MM14 x *B. megaterium* CCT 7730 (L2); MMM14, MMC3 and MMC14; bonferroni means st

Bartlett's test for equal variances: chi2(3) = 0.6557 Prob>chi2 = 0.884

L1 X L2	Summary of peroxide ((mMol de H2O2/106 U.F.C.) Mean	Std. Dev.
L1 MM14	1.324414	.10291232
L2 MMM14	1.3001574	.05488658
L2 MMC3	5.8216	.08232999
L2 MMC14	1.484508	.04116491
Total	2.4826698	2.0629876

Analysis of Variance

Source	SS	df	MS	F	Prob > F
Between groups	29.7693475	3	9.92311584	1797.97	0.0000
Within groups	.022076259	4	.005519065		
Total	29.7914238	7	4.25591768		

Comparison of peroxide ((mMol de H2O2/106 U.F.C.) by *B. megaterium* CCT 7729 (L1); MM14 x *B. megaterium* CCT 7730 (L2); MMM14, MMC3 and MMC14

(Bonferroni)

Row Mean- Col Mean	L1 MM14	L2 MMM14	L2 MMC3
L2 MMM14	-.024257		
	1.000		
L2 MMC3	4.49719	4.52144	
	0.000	0.000	
L2 MMC14	.160094	.184351	-4.33709
	0.585	0.409	0.000

Section 3.7

Peroxide; *B. megaterium* CCT 7729 (L1);_MMM3 X *B. megaterium* CCT 7730 (L2); MM3, MM14 and MMM3; bonferroni means st

Bartlett's test for equal variances: chi2(3) = 1.3454 Prob>chi2 = 0.718

L1 X L2	Summary of peroxide ((mMol de H2O2/106 U.F.C.) Mean	Std. Dev.
L1 MMM3	.69859198	.08232986
L2 MM3	.33474199	.02058248
L2 MM14	1.4133551	.09015305
L2 MMM3	.98967203	.08232986
Total	.92067526	.4406294

Analysis of Variance

Source	SS	df	MS	F	Prob > F
Between groups	1.52299897	3	.507666324	83.95	0.0001
Within groups	.030235196	5	.006047039		
Total	1.55323417	8	.194154271		

Comparison of peroxide ((mMol de H2O2/106 U.F.C.) by *B. megaterium* CCT 7729 (L1);_MMM3 X *B. megaterium* CCT 7730 (L2); MM3, MM14 and MMM3

(Bonferroni)

Row Mean-Col Mean	L1 MMM3	L2 MM3	L2 MM14
L2 MM3	-.36385		
	0.033		
L2 MM14	.714763	1.07861	
	0.001	0.000	
L2 MMM3	.29108	.65493	-.423683
	0.080	0.002	0.011

Section 3.8

Peroxide; *B. megaterium* CCT 7729 (L1);_MMM3 X *B. megaterium* CCT 7730 (L2); MMM14, MMC3 and MMC14; bonferroni means st

Bartlett's test for equal variances: chi2(3) = 0.4354 Prob>chi2 = 0.933

L1 X L2	Summary of peroxide ((mMol de H2O2/106 U.F.C.) Mean	Std. Dev.
L1 MMM3	.69859198	.08232986
L2 MMM14	1.3001574	.05488658
L2 MMC3	5.8216	.08232999
L2 MMC14	1.484508	.04116491
Total	2.3262143	2.1802536

Analysis of Variance

Source	SS	df	MS	F	Prob > F
Between groups	33.2562754	3	11.0854251	2427.88	0.0000
Within groups	.01826352	4	.00456588		
Total	33.2745389	7	4.75350556		

Comparison of peroxide ((mMol de H2O2/106 U.F.C.) by *B. megaterium* CCT 7729 (L1);_MMM3 X *B. megaterium* CCT 7730 (L2); MMM14, MMC3 and MMC14

(Bonferroni)

Row Mean-Col Mean	L1 MMM3	L2 MMM14	L2 MMC3
L2 MMM14	.601565		
	0.005		
L2 MMC3	5.12301	4.52144	
	0.000	0.000	
L2 MMC14	.785916	.184351	-4.33709
	0.002	0.315	0.000

Section 3.9

Peroxide; *B. megaterium* CCT 7729 (L1); MMM14 X *B. megaterium* CCT 7730 (L2); MM3, MM14 and MMM3; bonferroni means st

Bartlett's test for equal variances: chi2(3) = 1.8770 Prob>chi2 = 0.598

L1 X L2	Summary of peroxide ((mMol de H2O2/106 U.F.C.) Mean	Std. Dev.
L1 MMM14	.28622867	.0343041
L2 MM3	.33474199	.02058248
L2 MM14	1.4133551	.09015305
L2 MMM3	.98967203	.08232986
Total	.82903897	.52183101

Analysis of Variance

Source	SS	df	MS	F	Prob > F
Between groups	2.15382706	3	.717942355	145.72	0.0000
Within groups	.02463376	5	.004926752		
Total	2.17846082	8	.272307603		

Comparison of peroxide ((mMol de H2O2/106 U.F.C.) by *B. megaterium* CCT 7729 (L1); MMM14 X *B. megaterium* CCT 7730 (L2); MM3, MM14 and MMM3

(Bonferroni)

```
Row Mean-|

Col Mean |   L1 MMM14    L2 MM3     L2 MM14

---------+---------------------------------

  L2 MM3 |    .048513

         |      1.000

         |

 L2 MM14 |    1.12713    1.07861

         |      0.000      0.000

         |

 L2 MMM3 |    .703443     .65493   -.423683

         |      0.001      0.001      0.007
```

Section 3.10

Peroxide; *B. megaterium* CCT 7729 (L1); MMM14 X *B. megaterium* CCT 7730 (L2);_ MMM14, MMC3 and MMC14; bonferroni means st

Bartlett's test for equal variances: chi2(3) = 0.6371 Prob>chi2 = 0.888

L1 X L2	Summary of peroxide ((mMol de H2O2/106 U.F.C.) Mean	Std. Dev.
L1 MMM14	.28622867	.0343041
L2 MMM14	1.3001574	.05488658
L2 MMC3	5.8216	.08232999
L2 MMC14	1.484508	.04116491
Total	2.2231235	2.2743504

Analysis of Variance

Source	SS	df	MS	F	Prob > F
Between groups	36.1960276	3	12.0653425	3811.49	0.0000
Within groups	.012662085	4	.003165521		
Total	36.2086896	7	5.17266995		

Comparison of peroxide ((mMol de H2O2/106 U.F.C.) by *B. megaterium* CCT 7729 (L1); MMM14 X *B. megaterium* CCT 7730 (L2);_ MMM14, MMC3 and MMC14

(Bonferroni)

Row Mean-Col Mean	L1 MMM14	L2 MMM14	L2 MMC3
L2 MMM14	1.01393		
	0.000		
L2 MMC3	5.53537	4.52144	
	0.000	0.000	
L2 MMC14	1.19828	.184351	-4.33709
	0.000	0.184	0.000

Section 3.11

Peroxide; *B. megaterium* CCT 7729 (L1); MMC3 X *B. megaterium* CCT 7730 (L2);_MM3, MM14 and MMM3; bonferroni means st

Bartlett's test for equal variances: chi2(3) = 1.6863 Prob>chi2 = 0.640

L1 X L2	Summary of peroxide ((mMol de H2O2/106 U.F.C.) Mean	Std. Dev.
L1 MMC3	3.871364	.04116483
L2 MM3	.33474199	.02058248
L2 MM14	1.4133551	.09015305
L2 MMM3	.98967203	.08232986
Total	1.6257357	1.3411117

Analysis of Variance

Source	SS	df	MS	F	Prob > F
Between groups	14.3634929	3	4.78783097	951.80	0.0000
Within groups	.025151532	5	.005030306		
Total	14.3886444	8	1.79858056		

Comparison of peroxide ((mMol de H2O2/106 U.F.C.) by *B. megaterium* CCT 7729 (L1); MMC3 X *B. megaterium* CCT 7730 (L2);_MM3, MM14 and MMM3

(Bonferroni)

Row Mean- Col Mean	L1 MMC3	L2 MM3	L2 MM14
L2 MM3	-3.53662		
	0.000		
L2 MM14	-2.45801	1.07861	
	0.000	0.000	
L2 MMM3	-2.88169	.65493	-.423683
	0.000	0.002	0.007

Section 3.12

Peroxide; *B. megaterium* CCT 7729 (L1); MMC3 X *B. megaterium* CCT 7730 (L2); MMM14, MMC3 and MMC14; bonferroni means st

Bartlett's test for equal variances: chi2(3) = 0.4929 Prob>chi2 = 0.920

L1 X L2	Summary of peroxide (mMol de H2O2/106 U.F.C.) Mean	Std. Dev.
L1 MMC3	3.871364	.04116483
L2 MMM14	1.3001574	.05488658
L2 MMC3	5.8216	.08232999
L2 MMC14	1.484508	.04116491
Total	3.1194073	1.9897193

Analysis of Variance

Source	SS	df	MS	F	Prob > F
Between groups	27.6997003	3	9.23323344	2802.23	0.0000
Within groups	.013179857	4	.003294964		
Total	27.7128802	7	3.95898288		

Comparison of peroxide ((mMol de H_2O_2/106 U.F.C.) by *B. megaterium* CCT 7729 (L1); MMC3 X *B. megaterium* CCT 7730 (L2); MMM14, MMC3 and MMC14

(Bonferroni)

Row Mean-Col Mean	L1 MMC3	L2 MMM14	L2 MMC3
L2 MMM14	-2.57121		
	0.000		
L2 MMC3	1.95024	4.52144	
	0.000	0.000	
L2 MMC14	-2.38686	.184351	-4.33709
	0.000	0.195	0.000

Section 3.13

Peroxide; *B. megaterium* CCT 7729 (L1); MMC14 X *B. megaterium* CCT 7730 (L2); MM3, MM14 and MMM3; bonferroni means st

Bartlett's test for equal variances: chi2(3) = 3.0681 Prob>chi2 = 0.381

L1 X L2	Summary of peroxide ((mMol de H2O2/106 U.F.C.) Mean	Std. Dev.
L1 MMC14	1.193428	.01372167
L2 MM3	.33474199	.02058248
L2 MM14	1.4133551	.09015305
L2 MMM3	.98967203	.08232986
Total	1.0306388	.43127175

Analysis of Variance

Source	SS	df	MS	F	Prob > F
Between groups	1.46431728	3	.488105759	103.21	0.0001
Within groups	.023645273	5	.004729055		
Total	1.48796255	8	.185995319		

Comparison of peroxide ((mMol de H2O2/106 U.F.C.) by *B. megaterium* CCT 7729 (L1); MMC14 X *B. megaterium* CCT 7730 (L2); MM3, MM14 and MMM3

(Bonferroni)

Row Mean-Col Mean	L1 MMC14	L2 MM3	L2 MM14
L2 MM3	-.858686		
	0.000		
L2 MM14	.219927	1.07861	
	0.103	0.000	
L2 MMM3	-.203756	.65493	-.423683
	0.188	0.001	0.007

Section 3.14

Peroxide; *B. megaterium* CCT 7729 (L1); MMC14 X *B. megaterium* CCT 7730 (L2); MMM14, MMC3 and MMC14; bonferroni means st

Bartlett's test for equal variances: chi2(3) = 1.7012 Prob>chi2 = 0.637

L1 X L2	Summary of peroxide (mMol de H2O2/106 U.F.C.) Mean	Std. Dev.
L1 MMC14	1.193428	.01372167
L2 MMM14	1.3001574	.05488658
L2 MMC3	5.8216	.08232999
L2 MMC14	1.484508	.04116491
Total	2.4499233	2.084419

Analysis of Variance

Source	SS	df	MS	F	Prob > F
Between groups	30.4019446	3	10.1339815	3472.45	0.0000
Within groups	.011673598	4	.002918399		
Total	30.4136182	7	4.34480259		

Comparison of peroxide ((mMol de H2O2/106 U.F.C.) by *B. megaterium* CCT 7729 (L1) MMC14 X *B. megaterium* CCT 7730 (L2) MMM14, MMC3 and MMC14

(Bonferroni)

Row Mean-Col Mean	L1 MMC14	L2 MMM14	L2 MMC3
L2 MMM14	.106729		
	0.716		
L2 MMC3	4.62817	4.52144	
	0.000	0.000	
L2 MMC14	.29108	.184351	-4.33709
	0.034	0.162	0.000
