# Supplementary material for: Bacillus megaterium strains derived from water and soil exhibit differential responses to the herbicide mesotrione
Source: PLoS One. 2018 Apr 25;13(4):e0196166. doi: 10.1371/journal.pone.0196166 (PMC5918998; doi:10.1371/journal.pone.0196166)
Supplement: S4 File — Statistical analyzes. (PDF) [file pone.0196166.s004.pdf]

S4 File. MDA.

Section 4.1

MDA; *B. megaterium* CCT 7729 (L1); MM, MMM and MMC; 3 and 14h; bonferroni means st

Bartlett's test for equal variances: chi2(5) = 4.2097 Prob>chi2 = 0.520

L1	Summary of MDA (nmol/fresh weight) Mean	Std. Dev.
L1 MM3	1.7825	.10960153
L1 MM14	.43916667	.04474465
L1 MMM3	2.2475001	.10960153
L1 MMM14	.77500001	.10960157
L1 MMC3	2.17	.32880459
L1 MMC14	.27124999	.16440232
Total	1.2161539	.85389892

Analysis of Variance

Source	SS	df	MS	F	Prob > F
Between groups	8.57453807	5	1.71490761	68.52	0.0000
Within g	.175182246	7	.025026035		
Total	8.74972032	12	.72914336		

Comparison of MDA (nmol/fresh weight) by *B. megaterium* CCT 7729 (L1); MM, MMM and MMC; 3 and 14h

(Bonferroni)

Row Mean-Col Mean	L1 MM3	L1 MM14	L1 MMM3	L1 MMM14	L1 MMC3
L1 MM14	-1.34333				
	0.001				
L1 MMM3	.465	1.80833			
	0.326	0.000			
L1 MMM14	-1.0075	.335833	-1.4725		
	0.006	0.794	0.001		
L1 MMC3	.3875	1.73083	-.0775	1.395	
	0.662	0.000	1.000	0.001	
L1 MMC14	-1.51125	-.167917	-1.97625	-.50375	-1.89875
	0.000	1.000	0.000	0.231	0.000

Section 4.2

MDA; *B. megaterium* CCT 7730 (L2); MM, MMM and MMC; 3 and 14h; bonferroni means st

Bartlett's test for equal variances: chi2(4) = 0.7066 Prob>chi2 = 0.951

L2	Summary of MDA (nmol/fresh weight) Mean	Std. Dev.
L2 MM3	1.55	0
L2 MM14	1.395	.10960153
L2 MMM3	.89125001	.05480077
L2 MMM14	1.705	.10960153
L2 MMC3	.50375001	.05480079
L2 MMC14	1.3175	.10960153
Total	1.2270833	.43192333

Analysis of Variance

Source	SS	df	MS	F	Prob > F
Between groups	2.01009162	5	.402018324	57.37	0.0001
Within groups	.042043737	6	.007007289		
Total	2.05213536	11	.18655776		

Comparison of MDA (nmol/fresh weight) by *B. megaterium* CCT 7730 (L2); MM, MMM and MMC; 3 and 14h

(Bonferroni)

Row Mean- Col Mean	L2 MM3	L2 MM14	L2 MMM3	L2 MMM14	L2 MMC3
L2 MM14	-.155				
	1.000				
L2 MMM3	-.65875	-.50375			
	0.003	0.014			
L2 MMM14	.155	.31	.81375		
	1.000	0.151	0.001		
L2 MMC3	-1.04625	-.89125	-.3875	-1.20125	
	0.000	0.001	0.054	0.000	
L2 MMC14	-.2325	-.0775	.42625	-.3875	.81375
	0.482	1.000	0.034	0.054	0.001

Section 4.3

MDA; *B. megaterium* CCT 7729 (L1); MM3 X *B. megaterium* CCT 7730 (L2); MM3, MM14 and MMM3; bonferroni means st

Bartlett's test for equal variances: chi2(2) = 0.3622 Prob>chi2 = 0.834

L1 x L2	Summary of MDA (nmol/fresh weight) Mean	Std. Dev.
L1 MM3	1.7825	.10960153
L2 MM3	1.55	0
L2 MM14	1.395	.10960153
L2 MMM3	.89125001	.05480077
Total	1.4046875	.35499858

Analysis of Variance

Source	SS	df	MS	F	Prob > F
Between groups	.855139831	3	.28504661	42.19	0.0017
Within groups	.027028115	4	.006757029		
Total	.882167946	7	.126023992		

Comparison of MDA (nmol/fresh weight) by *B. megaterium* CCT 7729 (L1); MM3 X *B. megaterium* CCT 7730 (L2); MM3, MM14 and MMM3

(Bonferroni)

Row Mean- Col Mean	L1 MM3	L2 MM3	L2 MM14
L2 MM3	-.2325		
	0.285		
L2 MM14	-.3875	-.155	
	0.055	0.795	
L2 MMM3	-.89125	-.65875	-.50375
	0.002	0.008	0.022

Section 4.4

MDA; *B. megaterium* CCT 7729 (L1); MM3 X *B. megaterium* CCT 7730 (L2); MMM14, MMC3 and MMC14; bonferroni means st

Bartlett's test for equal variances: chi2(3) = 0.3923 Prob>chi2 = 0.942

L1 x L2	Summary of MDA (nmol/fresh weight) Mean	Std. Dev.
L1 MM3	1.7825	.10960153
L2 MMM14	1.705	.10960153
L2 MMC3	.50375001	.05480079
L2 MMC14	1.3175	.10960153
Total	1.3271875	.54712633

Analysis of Variance

Source	SS	df	MS	F	Prob > F
Between groups	2.05638992	3	.685463308	70.23	0.0006
Within groups	.039040613	4	.009760153		
Total	2.09543054	7	.299347219		

Comparison of MDA (nmol/fresh weight) by *B. megaterium* CCT 7729 (L1); MM3 X *B. megaterium* CCT 7730 (L2); MMM14, MMC3 and MMC14

(Bonferroni)

Row Mean-Col Mean	L1 MM3	L2 MMM14	L2 MMC3
L2 MMM14	-.0775		
	1.000		
L2 MMC3	-1.27875	-1.20125	
	0.001	0.002	
L2 MMC14	-.465	-.3875	.81375
	0.056	0.103	0.007

Section 4.5

MDA; *B. megaterium* CCT 7729 (L1); MM14 X *B. megaterium* CCT 7730 (L2); MM3, MM14 and MMM3, bonferroni means st

Bartlett's test for equal variances: chi2(2) = 0.9184 Prob>chi2 = 0.632

L1 X L2	Summary of MDA (nmol/fresh weight) Mean	Std. Dev.
L1 MM14	.43916667	.04474465
L2 MM3	1.55	0
L2 MM14	1.395	.10960153
L2 MMM3	.89125001	.05480077
Total	.99888888	.48776451

Analysis of Variance

Source	SS	df	MS	F	Prob > F
Between groups	1.88429396	3	.628097987	165.12	0.0000
Within groups	.019019788	5	.003803958		
Total	1.90331375	8	.237914219		

Comparison of MDA (nmol/fresh weight) by *B. megaterium* CCT 7729 (L1); MM14 X *B. megaterium* CCT 7730 (L2); MM3, MM14 and MMM3

(Bonferroni)

Row Mean- Col Mean	L1 MM14	L2 MM3	L2 MM14
L2 MM3	1.11083		
	0.000		
L2 MM14	.955833	-.155	
	0.000	0.322	
L2 MMM3	.452083	-.65875	-.50375
	0.003	0.001	0.003

Section 4.6

MDA; *B. megaterium* CCT 7729 (L1); MM14 X *B. megaterium* CCT 7730 (L2); MMM14, MMC3 and MMC14; bonferroni means st

Bartlett's test for equal variances: chi2(3) = 1.2205 Prob>chi2 = 0.748

L1 X L2	Summary of MDA (nmol/fresh weight) Mean	Std. Dev.
L1 MM14	.43916667	.04474465
L2 MMM14	1.705	.10960153
L2 MMC3	.50375001	.05480079
L2 MMC14	1.3175	.10960153
Total	.93000001	.57213691

Analysis of Variance

Source	SS	df	MS	F	Prob > F
Between groups	2.58769282	3	.862564273	138.98	0.0000
Within groups	.031032286	5	.006206457		
Total	2.6187251	8	.327340638		

Comparison of MDA (nmol/fresh weight) by *B. megaterium* CCT 7729 (L1); MM14 X *B. megaterium* CCT 7730 (L2); MMM14, MMC3 and MMC14

(Bonferroni)

Row Mean-Col Mean	L1 MM14	L2 MMM14	L2 MMC3
L2 MMM14	1.26583		
	0.000		
L2 MMC3	.064583	-1.20125	
	1.000	0.000	
L2 MMC14	.878333	-.3875	.81375
	0.000	0.026	0.001

Section 4.7

MDA; *B. megaterium* CCT 7729 (L1); MMM3 X *B. megaterium* CCT 7730 (L2); MM3, MM14 and MMM3; bonferroni means st

Bartlett's test for equal variances: chi2(2) = 0.3622 Prob>chi2 = 0.834

L1 X L2	Summary of MDA (nmol/fresh weight) Mean	Std. Dev.
L1 MMM3	2.2475001	.10960153
L2 MM3	1.55	0
L2 MM14	1.395	.10960153
L2 MMM3	.89125001	.05480077
Total	1.5209375	.52225293

Analysis of Variance

Source	SS	df	MS	F	Prob > F
Between groups	1.88220874	3	.627402914	92.85	0.0004
Within groups	.027028115	4	.006757029		
Total	1.90923686	7	.272748122		

Comparison of MDA (nmol/fresh weight) by *B. megaterium* CCT 7729 (L1); MMM3 X *B. megaterium* CCT 7730 (L2); MM3, MM14 and MMM3

(Bonferroni)

Row Mean- Col Mean	L1 MMM3	L2 MM3	L2 MM14
L2 MM3	-.6975		
	0.006		
L2 MM14	-.8525	-.155	
	0.003	0.795	
L2 MMM3	-1.35625	-.65875	-.50375
	0.000	0.008	0.022

Section 4.8

MDA; *B. megaterium* CCT 7729 (L1); MMM3 X *B. megaterium* CCT 7730 (L2); MMM14, MMC3 and MMC14; bonferroni means st

Bartlett's test for equal variances: chi2(3) = 0.3923 Prob>chi2 = 0.942

L1 X L2	Summary of MDA (nmol/fresh weight) Mean	Std. Dev.
L1 MMM3	2.2475001	.10960153
L2 MMM14	1.705	.10960153
L2 MMC3	.50375001	.05480079
L2 MMC14	1.3175	.10960153
Total	1.4434375	.68312825

Analysis of Variance

Source	SS	df	MS	F	Prob > F
Between groups	3.2276088	3	1.0758696	110.23	0.0003
Within groups	.039040613	4	.009760153		
Total	3.26664941	7	.466664202		

Comparison of MDA (nmol/fresh weight) by *B. megaterium* CCT 7729 (L1); MMM3 X *B. megaterium* CCT 7730 (L2); MMM14, MMC3 and MMC14

(Bonferroni)

Row Mean-Col Mean	L1 MMM3	L2 MMM14	L2 MMC3
L2 MMM14	-.5425		
	0.032		
L2 MMC3	-1.74375	-1.20125	
	0.000	0.002	
L2 MMC14	-.93	-.3875	.81375
	0.004	0.103	0.007

Section 4.9

MDA; *B. megaterium* CCT 7729 (L1); MMM14 X *B. megaterium* CCT 7730 (L2); MM3, MM14 and MMM3; bonferroni means st

Bartlett's test for equal variances: chi2(2) = 0.3622 Prob>chi2 = 0.834

L1 X L2	Summary of MDA (nmol/fresh weight) Mean	Std. Dev.
L1 MMM14	.77500001	.10960157
L2 MM3	1.55	0
L2 MM14	1.395	.10960153
L2 MMM3	.89125001	.05480077
Total	1.1528125	.35499856

Analysis of Variance

Source	SS	df	MS	F	Prob > F
Between groups	.855139726	3	.285046575	42.19	0.0017
Within groups	.027028124	4	.006757031		
Total	.88216785	7	.126023979		

Comparison of MDA (nmol/fresh weight) by *B. megaterium* CCT 7729 (L1); MMM14 X *B. megaterium* CCT 7730 (L2); MM3, MM14 and MMM3

(Bonferroni)

Row Mean-Col Mean	L1 MMM14	L2 MM3	L2 MM14
L2 MM3	.775		
	0.004		
L2 MM14	.62	-.155	
	0.010	0.795	
L2 MMM3	.11625	-.65875	-.50375
	1.000	0.008	0.022

Section 4.10

MDA; *B. megaterium* CCT 7729 (L1); MMM14 X *B. megaterium* CCT 7730 (L2); MMM14, MMC3 and MMC14; bonferroni means st

Bartlett's test for equal variances: chi2(3) = 0.3923 Prob>chi2 = 0.942

L1 X L2	Summary of MDA (nmol/fresh weight) Mean	Std. Dev.
L1 MMM14	.77500001	.10960157
L2 MMM14	1.705	.10960153
L2 MMC3	.50375001	.05480079
L2 MMC14	1.3175	.10960153
Total	1.0753125	.5047072

Analysis of Variance

Source	SS	df	MS	F	Prob > F
Between groups	1.74406491	3	.581354971	59.56	0.0009
Within groups	.039040622	4	.009760156		
Total	1.78310554	7	.254729362		

Comparison of MDA (nmol/fresh weight) by *B. megaterium* CCT 7729 (L1); MMM14 X *B. megaterium* CCT 7730 (L2); MMM14, MMC3 and MMC14

(Bonferroni)

Row Mean-Col Mean	L1 MMM14	L2 MMM14	L2 MMC3
L2 MMM14	.93		
	0.004		
L2 MMC3	-.27125	-1.20125	
	0.310	0.002	
L2 MMC14	.5425	-.3875	.81375
	0.032	0.103	0.007

Section 4.11

MDA; *B. megaterium* CCT 7729 (L1); MMC3 X *B. megaterium* CCT 7730 (L2); MM3, MM14 and MMM3; bonferroni means st

Bartlett's test for equal variances: chi2(2) = 1.9904 Prob>chi2 = 0.370

	Summary of MDA (nmol/fresh weight)	
L1 X L2	Mean	Std. Dev.
L1 MMC3	2.17	.32880459
L2 MM3	1.55	0
L2 MM14	1.395	.10960153
L2 MMM3	.89125001	.05480077
Total	1.5015625	.50555649

Analysis of Variance

Source	SS	df	MS	F	Prob > F
Between groups	1.66598344	3	.555327814	18.04	0.0087
Within groups	.12312808	4	.03078202		
Total	1.78911152	7	.25558736		

Comparison of MDA (nmol/fresh weight) by *B. megaterium* CCT 7729 (L1); MMC3 X *B. megaterium* CCT 7730 (L2); MM3, MM14 and MMM3

(Bonferroni)

Row Mean-Col Mean	L1 MMC3	L2 MM3	L2 MM14
L2 MM3	-.62		
	0.145		
L2 MM14	-.775	-.155	
	0.069	1.000	
L2 MMM3	-1.27875	-.65875	-.50375
	0.011	0.119	0.272

Section 4.12

MDA; *B. megaterium* CCT 7729 (L1); MMC3 X *B. megaterium* CCT 7730 (L2); MMM14, MMC3 and MMC14; bonferroni means st

Bartlett's test for equal variances: chi2(3) = 2.3473 Prob>chi2 = 0.504

L1 X L2	Summary of MDA (nmol/fresh weight) Mean	Std. Dev.
L1 MMC3	2.17	.32880459
L2 MMM14	1.705	.10960153
L2 MMC3	.50375001	.05480079
L2 MMC14	1.3175	.10960153
Total	1.4240625	.66788569

Analysis of Variance

Source	SS	df	MS	F	Prob > F
Between groups	2.98735847	3	.995786158	29.47	0.0035
Within groups	.135140577	4	.033785144		
Total	3.12249905	7	.446071293		

Comparison of MDA (nmol/fresh weight) by *B. megaterium* CCT 7729 (L1); MMC3 X *B. megaterium* CCT 7730 (L2); MMM14, MMC3 and MMC14

(Bonferroni)

Row Mean- Col Mean	L1 MMC3	L2 MMM14	L2 MMC3
L2 MMM14	-.465		
	0.388		
L2 MMC3	-1.66625	-1.20125	
	0.005	0.017	
L2 MMC14	-.8525	-.3875	.81375
	0.058	0.616	0.069

Section 4.13

MDA; *B. megaterium* CCT 7729 (L1); MMC14 X *B. megaterium* CCT 7730 (L2); MM3, MM14 and MMM3; bonferroni means st

Bartlett's test for equal variances: chi2(2) = 0.7185 Prob>chi2 = 0.698

	Summary of MDA (nmol/fresh weight)	
L1 X L2	Mean	Std. Dev.
L1 MMC14	.27124999	.16440232
L2 MM3	1.55	0
L2 MM14	1.395	.10960153
L2 MMM3	.89125001	.05480077
Total	1.026875	.53972503

Analysis of Variance

Source	SS	df	MS	F	Prob > F
Between groups	1.99707801	3	.665692669	63.33	0.0008
Within groups	.042043741	4	.010510935		
Total	2.03912175	7	.291303107		

Comparison of MDA (nmol/fresh weight) by *B. megaterium* CCT 7729 (L1); MMC14 X *B. megaterium* CCT 7730 (L2); MM3, MM14 and MMM3

(Bonferroni)

Row Mean- Col Mean	L1 MMC14	L2 MM3	L2 MM14
L2 MM3	1.27875		
	0.001		
L2 MM14	1.12375	-.155	
	0.002	1.000	
L2 MMM3	.62	-.65875	-.50375
	0.023	0.018	0.048

Section 4.14

MDA; *B. megaterium* CCT 7729 (L1); MMC14 X *B. megaterium* CCT 7730 (L2); MMM14, MMC3 and MMC14; bonferroni means st

Bartlett's test for equal variances: chi2(3) = 0.7387 Prob>chi2 = 0.864

L1 X L2	Summary of MDA (nmol/fresh weight) Mean	Std. Dev.
L1 MMC14	.27124999	.16440232
L2 MMM14	1.705	.10960153
L2 MMC3	.50375001	.05480079
L2 MMC14	1.3175	.10960153
Total	.94937501	.63063426

Analysis of Variance

Source	SS	df	MS	F	Prob > F
Between groups	2.72984074	3	.909946914	67.33	0.0007
Within groups	.054056239	4	.01351406		
Total	2.78389698	7	.397699569		

Comparison of MDA (nmol/fresh weight) by *B. megaterium* CCT 7729 (L1); MMC14 X *B. megaterium* CCT 7730 (L2); MMM14, MMC3 and MMC14

(Bonferroni)

Row Mean-Col Mean	L1 MMC14	L2 MMM14	L2 MMC3
L2 MMM14	1.43375		
	0.001		
L2 MMC3	.2325	-1.20125	
	0.697	0.003	
L2 MMC14	1.04625	-.3875	.81375
	0.005	0.174	0.013
