# Supplementary figures and images for: Bacillus megaterium strains derived from water and soil exhibit differential responses to the herbicide mesotrione
Source: PLoS One. 2018 Apr 25;13(4):e0196166. doi: 10.1371/journal.pone.0196166 (PMC5918998; doi:10.1371/journal.pone.0196166)

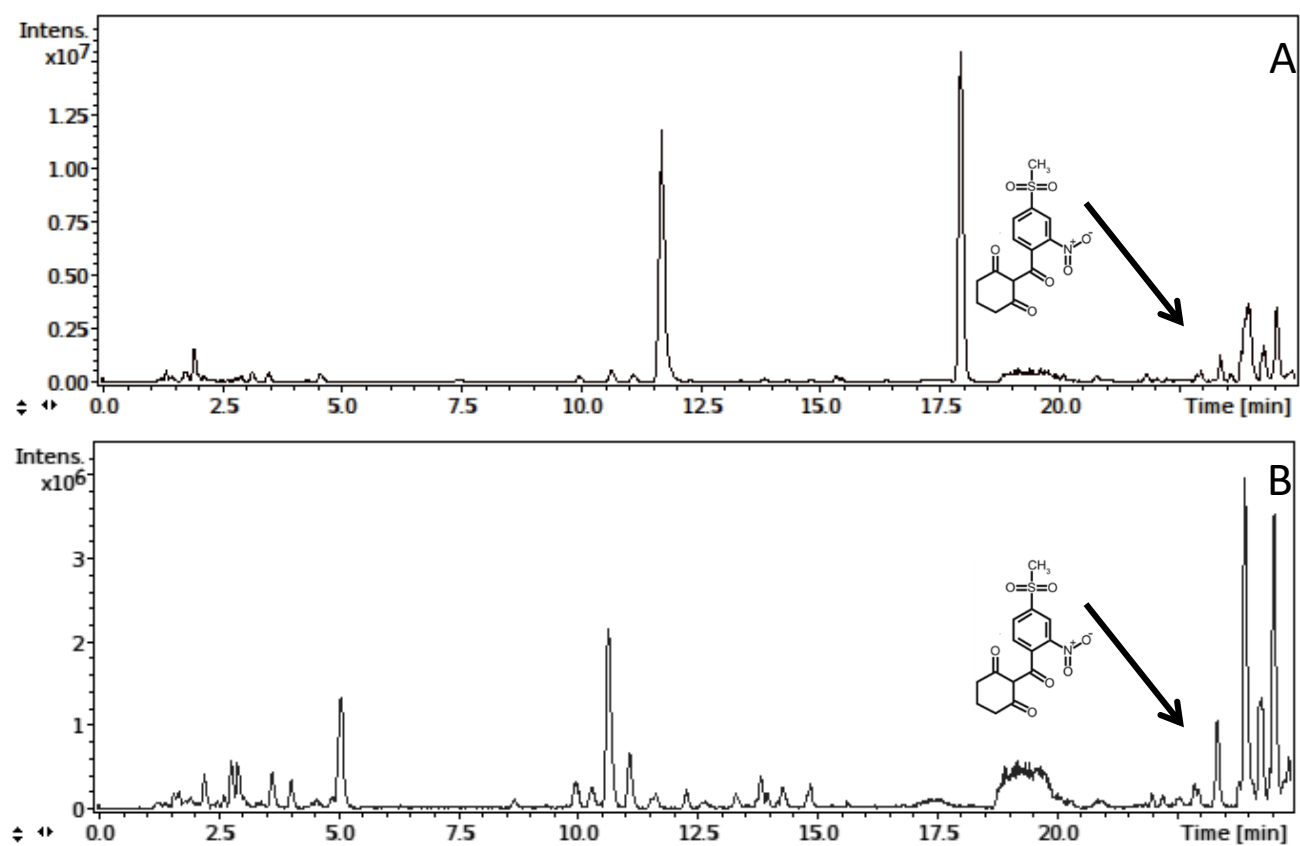

S1 Fig. Spectrogram 1.

Supplement: S1 Fig — A- LC-(+)ESIMS/MS analysis for mesotrione (Rt = 22.9 min.) added in the media at 24 h of incubation A- B. megaterium CCT 7729. B- B. megaterium CCT 7730. (PDF) [file pone.0196166.s005.pdf]

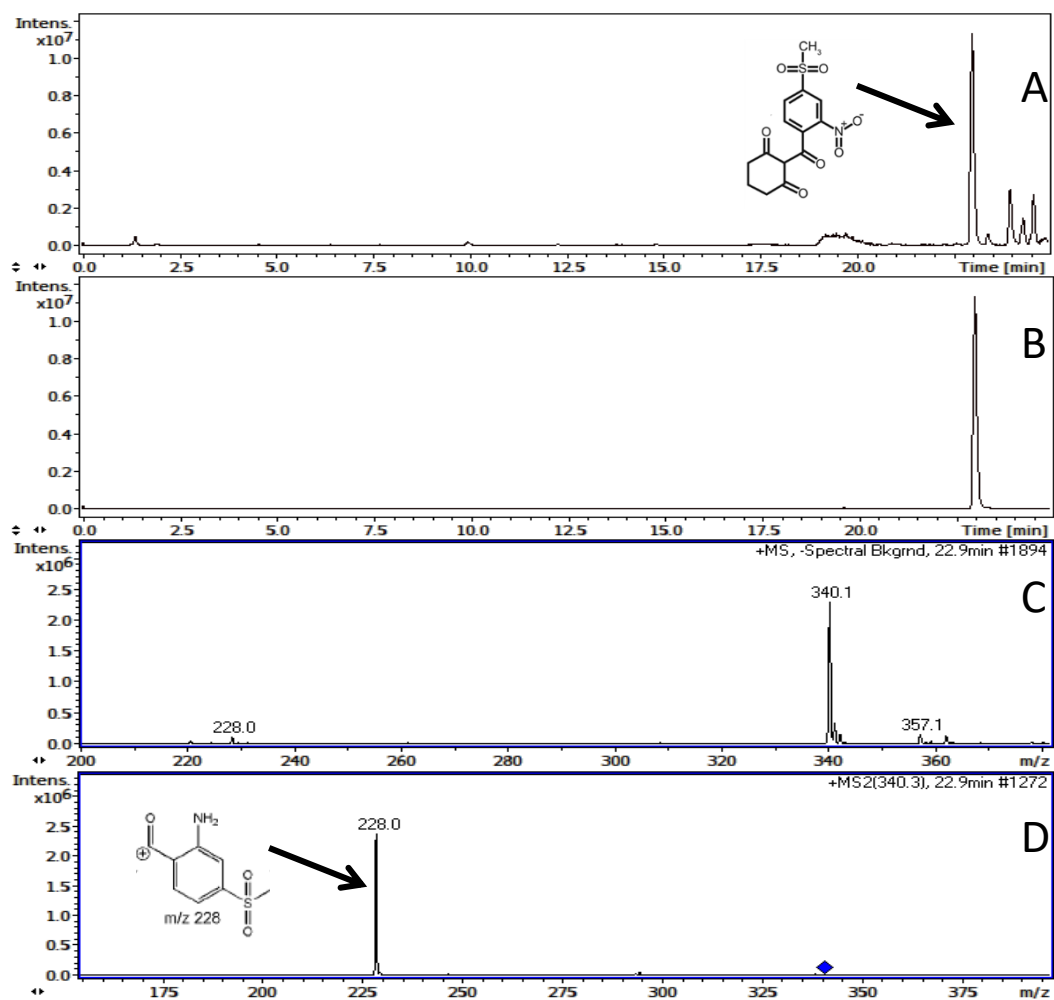

S2 Fig. Spectrogram 2.

Supplement: S2 Fig — A- LC-(+)ESIMS/MS analysis for mesotrione (Rt = 22.9 min.) added in the media at 0 h of incubation of B. megaterium CCT 7729. B- Extract peak chromatogram in Rt = 22.9 min. C- MS spectrum of peak in Rt = 22.9 min. D- LC-MS/MS spectrum of ion of m/z 340 [M+H]+. (PDF) [file pone.0196166.s006.pdf]

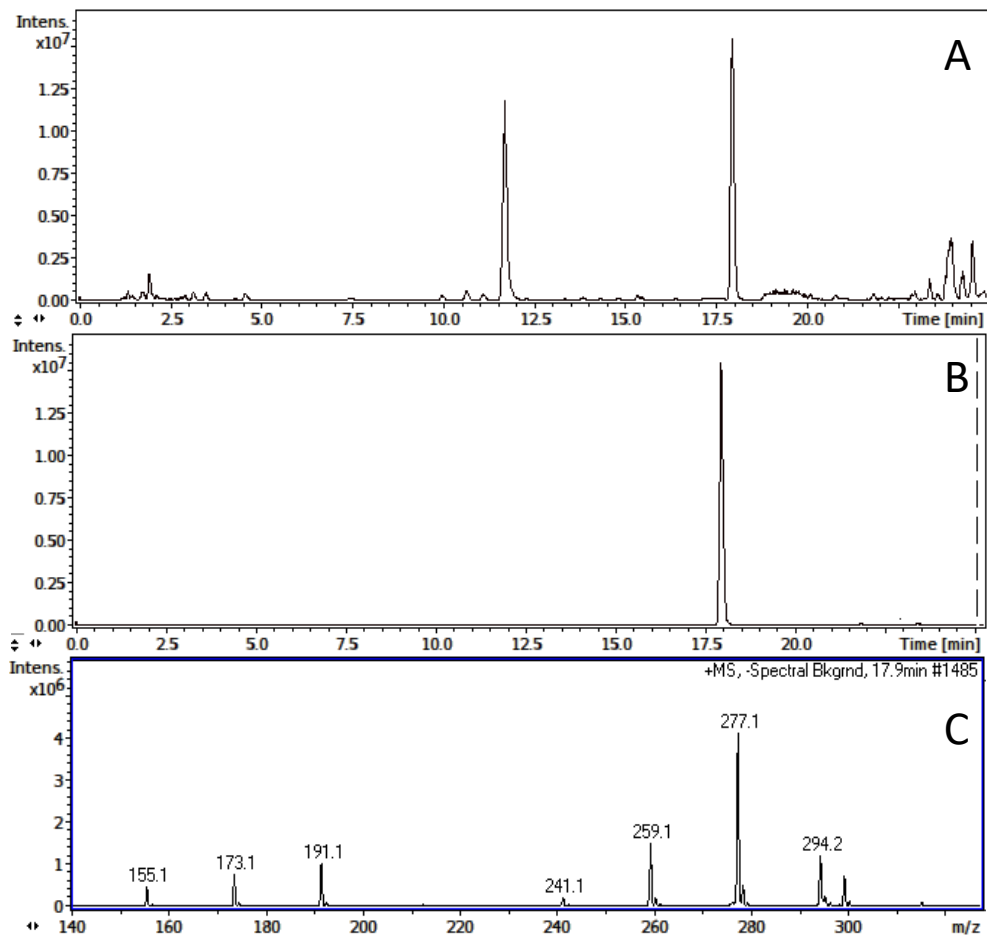

**S3 Fig. Spectrogram 3.**

Supplement: S3 Fig — A- LC-(+)ESIMS/MS analysis for mesotrione (Rt = 22.9 min) added in the media at 24 h of incubation of B. megaterium CCT 7729. B- Extract peak chromatogram in Rt = 17.8 min. C- MS spectrum of peak in Rt = 17.8 min. (PDF) [file pone.0196166.s007.pdf]

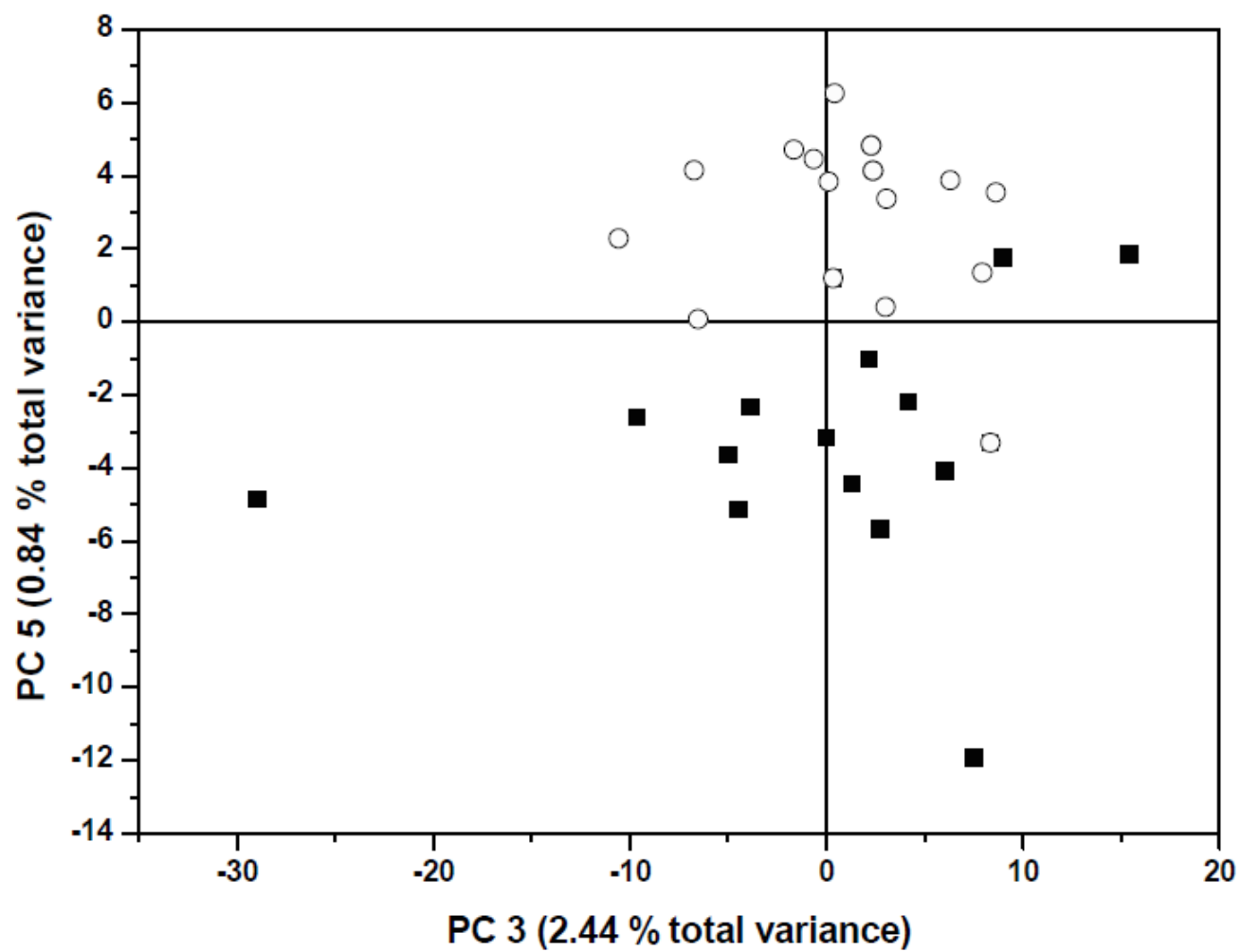

S4 Fig. PCA analysis.

Supplement: S4 Fig — PCA of the structure of lipid saturation of B. megaterium CCT 7729 (white spots) and B. megaterium CCT 7730 (dark spots) based on one million variables. PCA groups the data according to the degree of similarity. Samples with greater similarity are grouped in the same quadrant. (PDF) [file pone.0196166.s008.pdf]
