# Supplementary material for: Bacillus megaterium strains derived from water and soil exhibit differential responses to the herbicide mesotrione
Source: PLoS One. 2018 Apr 25;13(4):e0196166. doi: 10.1371/journal.pone.0196166 (PMC5918998; doi:10.1371/journal.pone.0196166)
Supplement: S1 Table — Quantitative analysis of infrared spectra of bands corresponding to alkanes C-H3 stretching (2956 and 2872 cm-1) and C-H2 bonds (2920 and 2850 cm-1); C = O stretching (1,750 cm-1); long chain hydrocarbons containing (-CH2-)n (1477–1452 cm-1 and 725 cm-1); -CH3 group indicative of the isopropyl group (1380 to 1350 cm-1); C-O group stretching vibration region of esters (1229 cm-1). Treatments (T column): 1–6—Bacillus megaterium CCT 7729; 7–12—Bacillus megaterium CCT 7730; 1–3 and 7–9–3 h of incubation; 4–6 and 10–12–14 h of incubation; 1, 4, 7 and 10 –MM; 2, 5, 8 and 11 –MMM; 3, 6, 9 and 12 –MMC. (PDF) [file pone.0196166.s010.pdf]

S1 Table. Quantitative analysis of infrared spectra.

T	CH_3/CH_2			C=O			$(-CH_2)n$		
	M	S	CV	M	S	CV	M	S	CV
**1**	42.56	2.77	6.51	13.37	1.43	10.70	16.47	1.02	6.21
**2**	42.47	2.94	6.92	14.87	2.37	15.94	15.07	1.40	9.29
**3**	42.67	1.39	3.26	13.23	1.13	8.55	14.89	0.84	5.66
**4**	41.11	3.53	8.58	15.48	0.96	6.17	16.22	2.02	12.48
**5**	40.78	3.98	9.63	14.97	1.87	12.46	14.42	0.50	3.46
**6**	46.14	3.56	7.70	14.62	0.87	5.93	15.81	1.00	6.35
**7**	40.50	1.38	3.41	13.54	1.24	9.12	14.82	1.64	11.04
**8**	42.99	3.34	7.59	12.92	1.05	8.10	14.54	1.43	9.87
**9**	40.32	1.88	4.66	14.32	1.08	7.52	14.97	0.27	1.77
**10**	42.30	3.31	7.83	13.99	2.14	15.26	14.24	0.73	5.14
**11**	37.49	2.90	7.74	16.03	0.55	3.41	14.51	0.55	3.77
**12**	40.76	0.59	1.44	13.21	1.37	10.34	14.78	1.00	6.79

	CH_3			C-O			$(CH_2)n$		
	M	S	CV	M	S	CV	M	S	CV
**1**	11.30	1.03	9.09	8.99	1.24	13.76	7.30	1.02	13.94
**2**	14.99	0.40	2.67	8.34	1.27	15.18	4.26	3.69	86.71
**3**	11.36	0.42	3.73	10.02	0.96	9.54	7.83	0.37	4.74
**4**	12.42	1.51	12.17	9.15	1.34	14.67	5.61	4.94	87.93
**5**	13.75	1.28	9.30	9.13	0.59	6.45	6.94	0.45	6.41
**6**	12.27	1.52	12.41	8.96	0.63	7.00	2.20	3.81	173.21
**7**	13.33	1.13	8.44	7.68	0.43	5.61	10.12	1.03	10.13
**8**	13.28	1.60	12.01	8.08	0.67	8.26	7.19	0.70	9.70
**9**	12.72	0.70	5.47	10.02	0.81	8.11	7.64	0.79	10.32
**10**	12.49	0.52	4.12	9.49	0.73	7.73	7.49	0.66	8.78
**11**	12.49	1.53	12.24	10.01	0.74	7.39	9.47	1.14	11.99
**12**	14.45	1.13	7.81	8.70	0.79	9.09	8.09	0.74	9.14
