# Supplementary material for: Bacillus megaterium strains derived from water and soil exhibit differential responses to the herbicide mesotrione
Source: PLoS One. 2018 Apr 25;13(4):e0196166. doi: 10.1371/journal.pone.0196166 (PMC5918998; doi:10.1371/journal.pone.0196166)
Supplement: S5 Fig — Regression model of vector FTIR spectrum extract in strains: (a) CCT 7729 and (b) CCT 7730. (PDF) [file pone.0196166.s009.pdf]

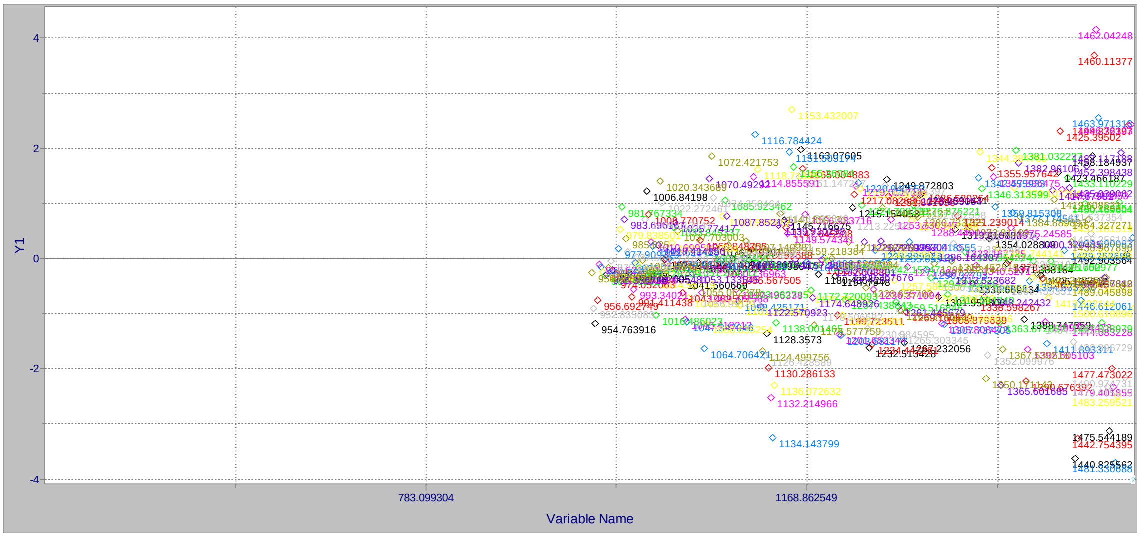

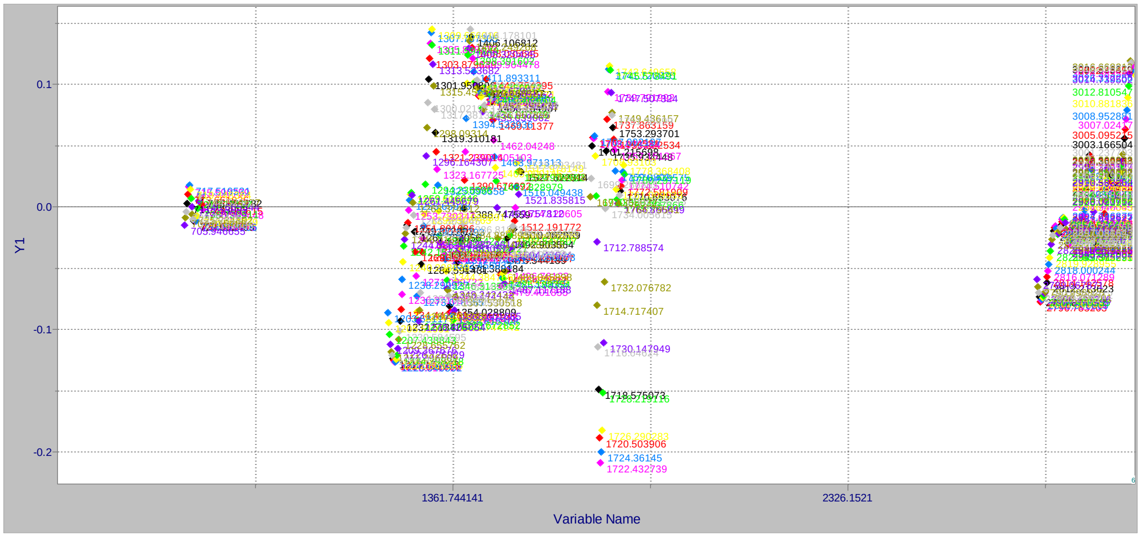

S5 Fig. Regression model.
